# *Drosophila melanogaster* cloak their eggs with pheromones, which prevents cannibalism

**DOI:** 10.1371/journal.pbio.2006012

**Published:** 2019-01-10

**Authors:** Sunitha Narasimha, Konstantin O. Nagornov, Laure Menin, Antonio Mucciolo, Astrid Rohwedder, Bruno M. Humbel, Martin Stevens, Andreas S. Thum, Yury O. Tsybin, Roshan K. Vijendravarma

**Affiliations:** 1 Department of Ecology and Evolution, University of Lausanne, Lausanne, Switzerland; 2 Spectroswiss Sàrl, EPFL Innovation Park, Lausanne, Switzerland; 3 Institute of Chemical Sciences and Engineering, EPFL, Lausanne, Switzerland; 4 Electron Microscopy Facility, University of Lausanne, Lausanne, Switzerland; 5 Department of Biology, University of Konstanz, Konstanz, Germany; 6 Centre for Ecology & Conservation, University of Exeter, United Kingdom; Cornell University, United States of America

## Abstract

Oviparous animals across many taxa have evolved diverse strategies that deter egg predation, providing valuable tests of how natural selection mitigates direct fitness loss. Communal egg laying in nonsocial species minimizes egg predation. However, in cannibalistic species, this very behavior facilitates egg predation by conspecifics (cannibalism). Similarly, toxins and aposematic signaling that deter egg predators are often inefficient against resistant conspecifics. Egg cannibalism can be adaptive, wherein cannibals may benefit through reduced competition and added nutrition, but since it reduces Darwinian fitness, the evolution of anticannibalistic strategies is rife. However, such strategies are likely to be nontoxic because deploying toxins against related individuals would reduce inclusive fitness. Here, we report how *D*. *melanogaster* use specific hydrocarbons to chemically mask their eggs from cannibal larvae. Using an integrative approach combining behavioral, sensory, and mass spectrometry methods, we demonstrate that maternally provisioned pheromone 7,11-heptacosadiene (7,11-HD) in the eggshell’s wax layer deters egg cannibalism. Furthermore, we show that 7,11-HD is nontoxic, can mask underlying substrates (for example, yeast) when coated upon them, and its detection requires pickpocket 23 (*ppk23*) gene function. Finally, using light and electron microscopy, we demonstrate how maternal pheromones leak-proof the egg, consequently concealing it from conspecific larvae. Our data suggest that semiochemicals possibly subserve in deceptive functions across taxa, especially when predators rely on chemical cues to forage, and stimulate further research on deceptive strategies mediated through nonvisual sensory modules. This study thus highlights how integrative approaches can illuminate our understanding on the adaptive significance of deceptive defenses and the mechanisms through which they operate.

## Author summary

Egg-laying species that lack parental care often protect their eggs from predators by laying them in communal groups or by fortifying them with toxins. However, these strategies may backfire when the predators are from the same species (cannibals) since a) there are plenty of available eggs in these sites, b) the cannibals may be resistant to the toxins, and c) poisoning cannibals who may be related would reduce inclusive fitness. Under these circumstances, natural selection should favor anticannibalistic strategies that are likely to be nontoxic. Here, we investigate how fruit flies (*Drosophila melanogaster*), which oviposit communally, protect their eggs from cannibalism by their own larvae. We show that maternal hydrocarbons incorporated into the egg’s wax layer to make them waterproof interestingly also serve as a mask that conceals their identity from cannibal larvae. In particular, we identify one female sex pheromone that deters cannibalism by forming a layer around the egg to conceal it. We further demonstrate that this pheromone is nontoxic and can mask underlying substrates such as yeast when used as a coating. While deceptive strategies (such as camouflage) deployed to avoid predation are extensively studied from a visual perspective, our findings suggest that deceptive strategies operating through other nonvisual sensory systems might be equally common across taxa.

## Introduction

Across most animal taxa, eggs are highly vulnerable to predators because they are immobile, highly nutritious, and defenseless. However, since egg production is costly [1], losing them to predation greatly reduces Darwinian fitness [2]. Animals have thus evolved several parent-modulated strategies: camouflage [3, 4], communal egg laying [5, 6], egg clustering [7], parental care [2], chemical defenses (toxins) [8, 9], and aposematic signaling [10] to mitigate this loss of fitness. On the other hand, eggs are not just vulnerable to interspecific predators but are equally at risk of predation from older conspecifics (cannibals), including parents and siblings [11–13]. Egg cannibalism is commonly dismissed as an aberrant behavior, resulting from unnatural breeding conditions. However, mounting evidence has demonstrated its adaptive value in several species, wherein the cannibals increase their fitness through both reduced competition and the supplemented nutrition [11, 14]. In support of this argument, egg cannibalism is common even among noncarnivorous species [15] and has also been shown to have important ecological consequences on population dynamics and stability [11, 16]. Nevertheless, egg cannibalism reduces direct fitness to parents and can additionally reduce inclusive fitness if the eggs consumed are genetically related to the cannibals [11]. Interestingly, most of the aforementioned parent-modulated strategies evolved in response to interspecific egg predators are often ineffective against conspecifics: while cannibals are generally resistant to conspecific toxins and aposematic signals [17], other strategies like producing surplus eggs and communal egg laying might even facilitate egg cannibalism [18, 19]. Additionally, since deploying toxic defenses against conspecifics would further reduce inclusive fitness [20], natural selection should favor the evolution of anticannibalistic strategies that are likely to be nontoxic.

Anticannibalistic strategies that deter egg cannibalism have evolved independently in species across taxa, and convincingly, none of them seem to be toxic. These strategies include laying of nondeveloping eggs (trophic eggs) within clutches by mothers to reduce cannibalism among offspring [11, 21], laying of eggs with protective coatings around the egg’s shell [22], laying eggs on specialized structures (like stalks) [23], nest guarding [24], and synchronized egg hatching [25]. Nevertheless, there are several other communally egg-laying species, including insects, that avoid cannibalizing eggs despite lacking the above strategies [15]. This prompted us to speculate about the existence of alternative strategies that could modulate this behavior in nature. The understanding of such strategies is crucial, especially to the fields of conservation, epidemiology, and pest management.

We recently reported predatory cannibalism among *D*. *melanogaster* larvae, wherein younger larval instars pack-hunt and consume older conspecific larvae under laboratory conditions [26]. Surprisingly, despite their predaceous nature, we never observed larvae attacking conspecific eggs, even upon starvation. In nature, *D*. *melanogaster* oviposit communally at sites already occupied by conspecific and heterospecific larvae to facilitate social feeding among larvae [27, 28]. Although these oviposition sites (decaying fruits) are nutritionally rich, they at times risk desiccation [27] and immense larval competition [29], both of which could coerce larvae to seek other food sources such as older conspecifics [26] and cadavers [30]. Interestingly, despite availability of several conspecific eggs in their vicinity, larvae never cannibalize them, either for food or for other benefits like reduced competition [15] and reduced risk of predation by younger larvae [26]. This observation thus raises important questions on why and how egg cannibalism is averted in this system.

**Fig 1:**
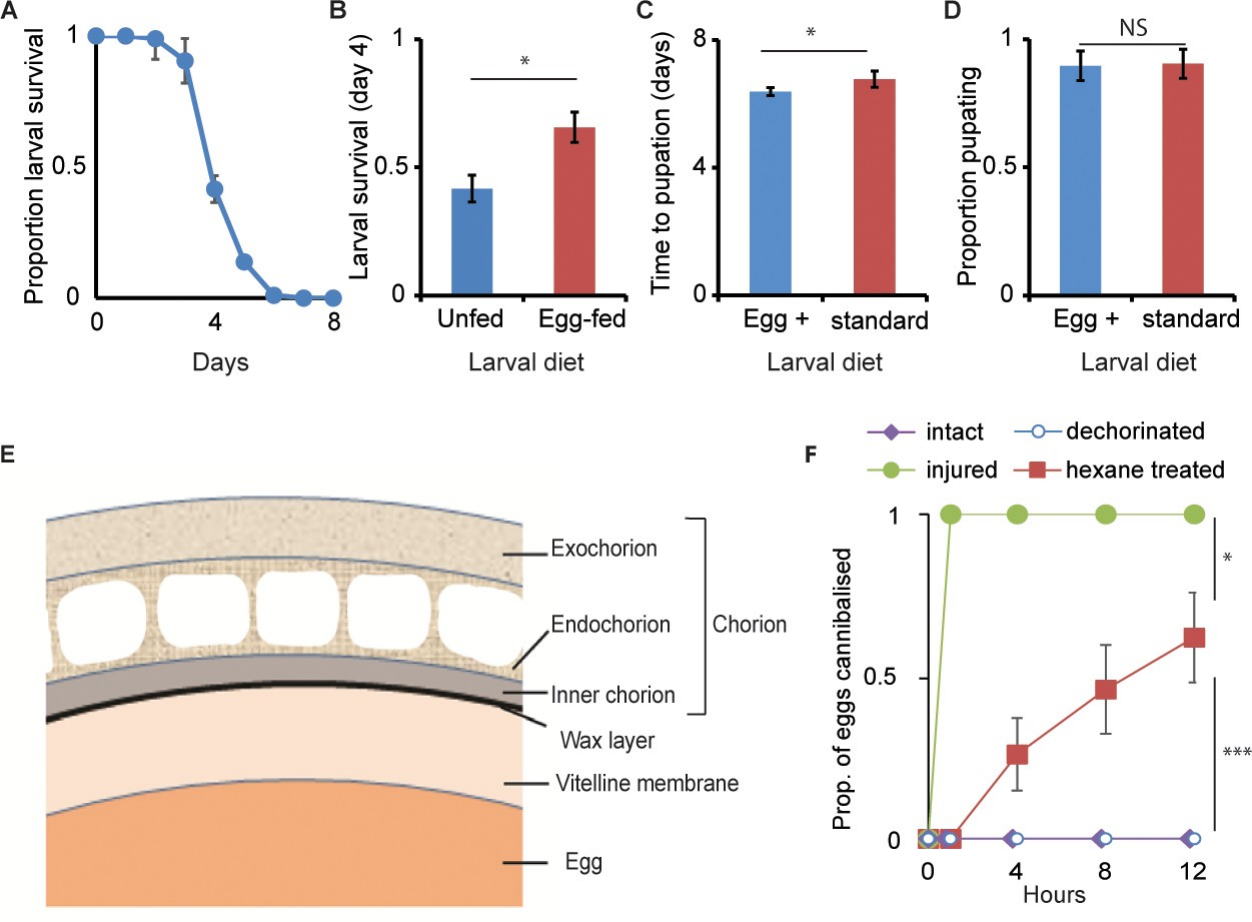
Wax layer of the eggshell prevents egg cannibalism. (A) Survival period of freshly hatched larvae held individually without food on agar (mean = 3.4 ± 0.1 days (SE); *n* = 4 replicate plates, 24 freshly hatched larvae assayed per replicate), demonstrating the surplus nutrition provisioned within an egg for embryonic development well beyond hatching. (B) Proportion of freshly hatched larvae surviving until day four, in absence of food (unfed) and when allowed to feed on two injured eggs (*n* = 4 replicate plates per treatment, 24 freshly hatched larvae assayed per replicate). Egg-fed larvae had a higher survival than unfed larvae (mean ± SD). (C, D) Larval developmental period (mean ± SD) and larval survival to pupation (mean ± SD), respectively, when reared on standard diet laced with (blue bars) or without (red bars) extract from crushed eggs (*n* = 4 bottles/diet, egg density: 200 eggs/bottle). On supplemented diet, larvae developed faster (ANOVA: *F*_*1*,*9*_ = 9.75, *p* = 0.014) but survived to a similar extent (ANOVA: *F*_*1*,*9*_ = 0.07, *p* = 0.8). (E) Graphical depiction of the egg shell morphology. The outer three layers—the exochorion, the endochorion, and the inner chorion layer—together form the chorion; the thin wax layer (in black) lies between the chorion and the innermost vitelline membrane that envelops the egg. (F) Proportion (mean ± SE) of intact, injured, dechorinated, and hexane-treated eggs cannibalized when confined in agar vials with second-instar larvae over 12 h (*n* = 10 vials/treatment, with 5 eggs and 10 larvae per vial). Larvae cannibalized hexane-treated eggs but not dechorinated eggs (ANOVA: *F*_*1*,*18*_ = 21.85, *p* = 0.0002), however, not to the extent of injured eggs (ANOVA: *F*_*1*,*18*_ = 7.57, *p* = 0.0132). Also see S1 Fig. ***p < 0.001, *p < 0.05. Data underlying this figure can be found in S1 Data. NS, not significant

Given that parental care in *D*. *melanogaster* is mostly limited to oviposition site selection and egg provisioning [31, 32], we hypothesized that parental provisioning in some form protects *D*. *melanogaster* eggs from conspecific larvae. Virgin *D*. *melanogaster* females can lay nonviable unfertilized eggs, equivalent to trophic eggs laid by other species. However, given that mated *D*. *melanogaster* seldom lay such unfertilized eggs, their production more likely represents a “risk–return strategy” (i.e., the cost of producing such unfertile eggs is less than what is risked by aborting them) rather than a strategy that mitigates cannibalism. In several insect species, the eggshell, its pigmentation and patterning, and specific extrachorionic modifications upon it are all known to deter predation [8]. *Drosophila* eggs are enveloped within a maternally provisioned eggshell during oogenesis that is composed of three distinct layers (Fig 1D): chorion, wax layer, and vitelline membrane, which serve to deter pathogens, prevent dehydration, and facilitate respiration, respectively [8]. However, the extent to which these eggshell layers play a role in deterring cannibals is not known.

In this study, we investigate the mechanisms that could deter egg cannibalism in *D*. *melanogaster*. To do this, we examine the protective role of the eggshell and its constitutive layers using an integrative approach. We consequently reveal a novel anticannibalistic strategy that is mediated through chemical deception and involves semiochemicals [33] present in the wax layer of the egg shell, a so-far overlooked strategy that could potentially be widespread across taxa.

## Results and discussion

### Wax layer prevents egg cannibalism by larvae

To understand the extent to which *D*. *melanogaster* females provision nutrients within their eggs, we assayed larval survival upon hatching in the absence of any food. The first-instar larvae survived for up to five days posthatching in the absence of food (Fig 1A), showing that the nutrients provisioned within an egg are surplus for embryonic development, and they can hence support larval survival well beyond hatching. Feeding two injured conspecific eggs to just-hatched larvae increased their survival (Fig 1B), confirming the previous finding that eggs are nutritious and are thus worth cannibalizing [30].

When egg consumption by second-instar larvae (food deprived for 2 h) was assayed, larvae surprisingly did not cannibalize intact viable eggs (Fig 1F). However, if the eggs presented to the larvae were injured (by pricking), the eggs were immediately consumed (Fig 1F), supporting the results from a recent study that used nonviable eggs [30]. The same larval response was observed when the eggs provided were from an unrelated *D*. *melanogaster* strain (panel D in S1 Fig). To rule out the possibility that the eggs are toxic to the larvae, we assayed larval development and survival on standard fly medium laced with crushed eggs. Compared to the control larvae raised on the standard diet, larvae supplemented with crushed eggs developed faster (Fig 1C) and survived equally well (Fig 1D), confirming that the eggs are nontoxic and nutritious.

Given that injured eggs were readily cannibalized (Fig 1F), we next tested whether breached eggshells facilitate cannibalism. We sequentially removed the two outer eggshell layers, the chorion membrane and the wax layer (Fig 1E), by treating the eggs with sodium hypochlorite and hexane, respectively [34, 35], and then presented these chemically treated eggs to food-deprived larvae. Indeed, removal of the thin wax layer (4–5 nm, hexane treated) but not the thick chorion layer (840–1,250 nm, dechorinated) [36] made 60% of eggs vulnerable to cannibals (Fig 1F). This refutes a recent report [30] that *D*. *melanogaster* larvae cannibalize dechorinated eggs, which we believe is due to the experimental procedure. The dechorinated eggs they use were killed prior to their assays by dyeing with tartrazine NaCl and storing them at 4°C. These treatments could have unintentionally damaged the eggs’ wax layer. In contrast, the consecutive treatment of eggs with sodium hypochlorite and hexane in our experiments had little or no effect on egg hatchability (panel A in S1 Fig), egg’s time to hatching (panel B in S1 Fig), and egg-to-adult viability (panel C in S1 Fig). The experiments above thus suggest that in addition to the primary role of the wax layer in preventing desiccation of the embryo [37], it might also serve to protect eggs from cannibal larvae.

### Wax-layer composition and parental role in its formation

**Fig 2:**
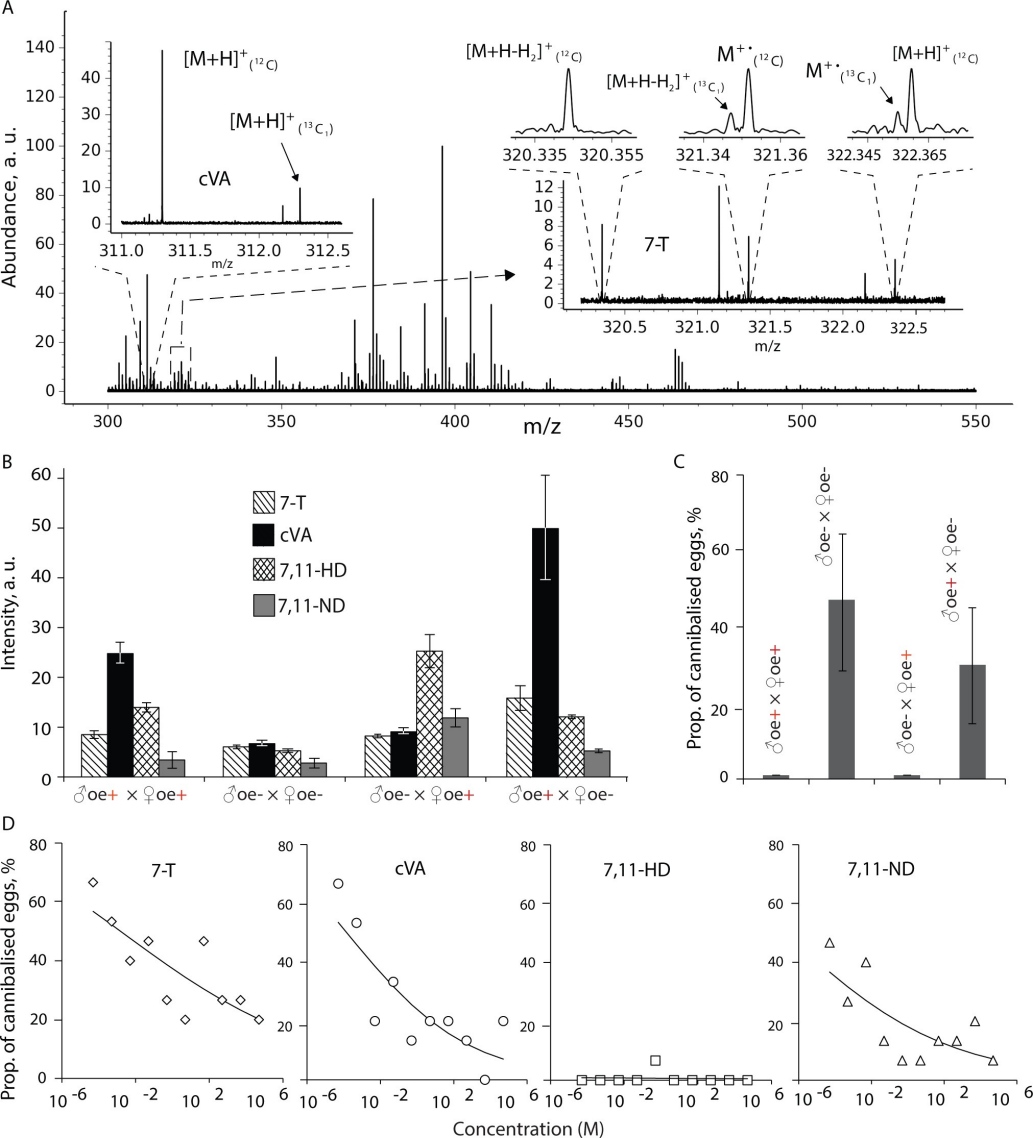
Maternally provisioned hydrocarbons confer protection. (A) Hydrocarbon profile of the wax layer in *D*. *melanogaster* eggs. (B) Relative amount (SNR) of four known sex-specific *D*. *melanogaster* pheromones (7-T, cVA, 7,11-HD, and 7,11-ND) present in the wax layer of eggs laid by the four *oe* mutant crosses. The relative amounts of pheromones were calculated from mass spectra acquired by high-resolution 10 T APPI-FT-ICR-MS of hexane extracts of wax layers (S2 Table). (C) Vulnerability of eggs laid by the four *oe* mutant crosses to larval cannibals, assayed as the proportion (mean ± SD) of these eggs cannibalized when confined with second-instar larvae (*n* = 7 replicate vials). Only eggs laid by the two crosses with *oe*^−^ mothers were cannibalized, to extents that only differed marginally (ANOVA: *F*_*1*,*12*_ = 3.66, *p* = 0.08). (D) Proportion (mean ± SE) of hexane-treated eggs that were cannibalized when perfumed with commercially synthesized 7-T, cVA, 7,11-HD, and 7,11-ND at different concentrations (*n* = 3 replicate vials/pheromone concentration, with 5 eggs and 10 larvae per vial). Least-squares means contrast of 7,11-HD versus other pheromones was different (*F*_*1*,*35*_ = 17.2, *p* = 0.0002). Also, see S2, S3 and S4 Figs. Data underlying this figure can be found in S1 Data. APPI FT-ICR MS, atmospheric pressure photoionization Fourier transform ion cyclotron resonance mass spectrometry; a.u., arbitrary units; cVa, 11-*cis*-vaccenyl acetate; *oe*, oenocyte; SNR, signal-to-noise ratio; 7-T, 7-tricoscene; 7,11-HD, 7,11-heptacosadiene; 7,11-ND, 7,11-nonacosadiene

Next, to understand the mechanism underlying the wax layer’s anticannibalistic function, we extracted this layer from fertilized eggs and analyzed its biochemical composition using gas chromatography hyphenated with mass spectrometry (GC-MS) and high-resolution atmospheric pressure photoionization Fourier transform ion cyclotron resonance mass spectrometry (APPI FT-ICR MS) [38]. We used these advanced mass analyzers with high accuracy (sub-parts–million mass accuracy) and resolving power, mainly to mine for low-molecular–weight compounds (for example, toxins). However, in addition, we aimed to establish methods that could unambiguously assign elemental formulas to metabolites for better characterization, especially in life sciences (see Materials and Methods). We identified 13 compounds (linear alkenes, alkadienes, and sterols) that were mostly known cuticular hydrocarbons (pheromones) of adult *D*. *melanogaster* [39] (Figs 2A and S2 and S1 Table), which are mainly known to be synthesized by specialized epicuticular cells called the “oenocytes” (*oe*) [39]. Below, we focus on four pheromones (7,11-heptacosadiene [7,11-HD]; 7,11-nonacosadiene [7,11-ND]; 7-tricoscene [7-T]; and 11-*cis*-vaccenyl acetate [cVA]), given that they are sex-specific, have known functions, and are commercially synthesizable.

The pheromone profile we detected in the wax layer was similar to the pheromones already known to be deposited by adult flies (both sexes) on egg-laying sites to facilitate aggregation [40, 41]. Furthermore, these pheromones are also known to be present in the reproductive tract of mated females [42]. Thus, to exclude the possibility of potential cross-contamination of our samples by adult flies, we analyzed the hexane washes of the outer chorion layer and that of dechorinated eggs and compared their hydrocarbon profiles. Most of the pheromones detected on the outer chorion (layer exposed to environment and female reproductive tract) were also found after dechorination (in the wax layer) (panel A in S3 Fig). The male pheromone cVA produced by the male’s ejaculatory bulb [43] was an exception; it was abundant only on the chorion but greatly reduced upon dechorination, suggesting that eggs acquire cVA postchoriogenesis either from the environment or from male ejaculate within the female reproductive tract [42] (panel A in S3 Fig). To further ascertain that these pheromones are indeed present in the wax layer, we analyzed three successive hexane washes of the dechorinated eggs and detected these pheromones at progressively decreasing concentrations across the washes, possibly reflecting the compact nature of the wax layer [37] (panel B in S3 Fig). Interestingly, the presence of such pheromones within the eggshell having other physiological functions has been previously reported in insects and nematodes [44, 45].

For over three decades, the wax layer has been considered to be synthesized by the follicle cells that surround the oocytes, exclusively based on electron microscopy observation of lipid endosomes within the follicle cells during oogenesis and their eventual deposition onto the vitelline membrane of the egg [46, 47]. Most of the hydrocarbons we detect in the wax layer are so far only known to be synthesized in the *oe*s [39]; for example, the biosynthesis of dienes like 7,11-HD and 7,11-ND (female-specific pheromones) requires the enzymatic action of a specific desaturase *desatF* (*Fad2*) in the *oe*s [48]. However, transcriptional data (microarray and RNA-sequencing [RNA-seq] data from FlyAtlas and FlyBase, respectively) show that this gene is not expressed in the ovary. Thus, further empirical investigations are necessary to clarify the role of follicle cells in the synthesis of the wax layer.

The presence of hydrocarbons specific to both sexes in the wax layer motivated us to track the parental origin of these pheromones using mutant flies with ablated *oe*s (*oe*^−^) [39]. Males and females with or without ablated *oe*s were crossed, and the wax-layer composition of their eggs was analyzed. The label-free, semiquantitatively established hydrocarbon profile of eggs appears to be directed by the cuticular hydrocarbon composition of the parental cross [39] they were laid by; eggs of *oe*^−^ parents had fewer hydrocarbons than those of *oe*^+^ parents (Fig 2B and S2 Table, and panel B in S4 Fig). The eggs parented by *oe*^+^ males and *oe*^−^ females had reduced female-specific hydrocarbons—for example, 7,11-HD and 7,11-ND—compared to male-specific hydrocarbons—for example, 7-T. Its reciprocal cross had reduced male-specific hydrocarbon (7-T) compared to the female-specific hydrocarbons. However, the reason as to why cVA, the male pheromone of non-*oe* origin, was also reduced in the eggs laid by this cross is unclear. The presence of hydrocarbons corresponding to *oe*^−^ parents at low concentrations in our samples could possibly be due to residual hydrocarbons produced prior to or during *oe* ablation. Thus, it seems that both parents contribute towards provisioning the pheromonal content of the wax layer. This suggests that wax-layer synthesis is likely to involve transportation of maternal and paternal hydrocarbons from the *oe*s and deposited seminal fluid, respectively, to the ovary during oogenesis. Nevertheless, the existence of such transport mechanisms involving lipophorin molecules has been speculated in *D*. *melanogaster* [31] and other insects [45].

### Maternal sex pheromone 7,11-HD deters egg cannibalism

The deterrent effect of hydrocarbons present on the egg surface towards cannibals has been previously speculated about in the coccinellid *Adalia bipunctata* [49]. However, our system allowed us to ascertain the deterrent role of sex-specific hydrocarbons in egg cannibalism. For this, we first assayed the vulnerability of eggs laid by the above four crosses to cannibal larvae. Conspicuously, larvae only cannibalized eggs with *oe*^−^ motherhood (Fig 2C). The eggs from the three *oe*^−^ mutant crosses had similar egg-to-adult viability (slightly less than eggs from the *oe*^+^ control), thus excluding nonviability of eggs with *oe*^−^ motherhood (panel E in S1 Fig). We next independently verified this deterrent role of hydrocarbons by assaying the vulnerability of hexane-washed eggs perfumed with four commercially synthesized hydrocarbons found most abundantly on the egg (7-T, cVA, 7,11-HD, and 7,11-ND; panel A in S4 Fig) to cannibal larvae. Since the actual concentration of the pheromones in the wax layer could not be determined (because of the differential solubility of the wax layer in hexane), the synthetic pheromones were applied and tested at several (serially diluted) concentrations. Interestingly, larvae only refrained from cannibalizing eggs that were perfumed with the female pheromone 7,11-HD (Fig 2D), even when present at very low concentrations. However, the other female pheromone 7,11-ND we used, despite being structurally very similar to 7,11-HD (with just two additional carbon atoms), failed to deter cannibalism (Fig 2D). Given that a) the eggs of male *oe*^+^ and female *oe*^−^ with 7,11-HD levels slightly lower than *oe*^+^ controls become vulnerable to cannibalism and b) eggs perfumed with various concentrations of 7,11-HD remain protected, these results strongly suggest that the deterrent function of 7,11-HD is dose independent. These findings suggest that 7,11-HD, rather than the overall wax layer, deters egg cannibalism; nevertheless, the synergistic effect of other hydrocarbons and chemicals we detect within the wax layer (S2 Fig and S1 Table) cannot be ruled out.

### Response of larval sensory receptors to 7,11-HD

**Fig 3:**
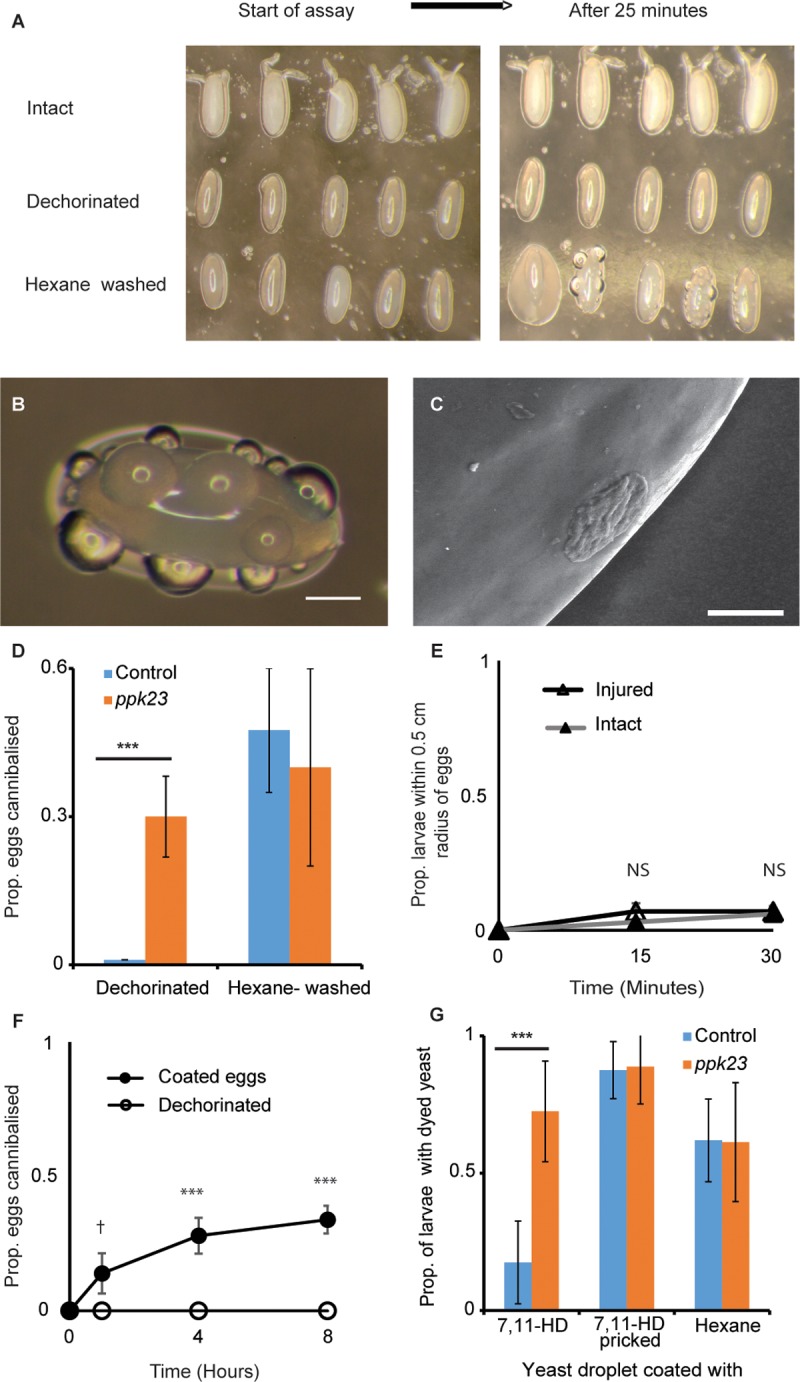
Maternal hydrocarbons form a waterproof layer that prevents leakage of eggs. (A) Egg contents leaking out of hexane-washed eggs but not from dechorinated or intact eggs when allowed to stand on an agar surface for over 25 min (*n* = 10 eggs/treatment). (B) Egg contents leak through the vitelline membrane in the form of tiny droplets; bar = 100 μm. (C) Cryo-electron scanning of an egg leak showing egg contents permeating through the vitelline membrane; bar = 20 μm. (D) Proportion (mean ± SD) of dechorinated (dark bars) and hexane-washed (light bars) eggs cannibalized by Canton S and *ppk23* mutant larvae (ANOVA: *n* = 4 replicates, 10 eggs/replicate). (E) Larvae do not differentiate injured (open triangle) and intact (closed triangle) eggs in a simple choice assay and do not get attracted to injured eggs (*n* = 5 replicate plates; ANOVA, *F*_*1*,*8*_ = 0.78, *p* = 0.404). (F) Larvae cannibalize only dechorinated eggs coated with egg contents of injured eggs (closed circle) but not dechorinated eggs (open circle); (mean ± SE; *n* = 5 replicate vials/treatment; ANOVA, *F*_*1*,*8*_ = 129.2, *p* < 0.0001). (G) Larval feeding (mean ± SD) is significantly reduced when yeast droplets are coated with 7,11-HD than when coated with the solvent alone (hexane); feeding is, however, completely restored when the 7,11-HD layer is pricked (*n* = 8 replicate Petri plates/treatment). Furthermore, 7,11-HD coated on yeast droplets does not inhibit feeding in *ppk23* mutant larvae (mean ± SD) and is not different than when coated with solvent alone (hexane). However, feeding on yeast is further facilitated when the 7,11-HD layer is pricked (ANOVA; *n* = 8 replicate Petri plates/treatment). Also see S6 and S7 Figs. ***p < 0.0001, **p < 0.001, †*p* < 0.1. Data underlying this figure can be found in S1 Data. NS, not significant; *ppk23*, pickpocket 23; 7,11-HD, 7,11-heptacosadiene

Next, we attempted to identify the larval sensory receptors associated with this 7,11-HD–mediated deterrence effect. In adult *D*. *melanogaster*, 7,11-HD mediates mate recognition [39], maintains the species barrier with *D*. *simulans* [50], and is used to mark sites suitable for mating and oviposition [51]. It was recently reported [52] that in *D*. *melanogaster*, 7,11-HD detection is dependent on the joined action of the three receptor genes pickpocket 23 (*ppk23*), *ppk25*, and *ppk29* that are necessary for the function of gustatory neurons located in their forelegs [53, 54]. However, whether and how larvae respond to 7,11-HD remains elusive [55]. Anticipating that the role of *ppk23* in 7,11-HD detection may be conserved in larvae, we tested whether egg cannibalism is promoted when *ppk23* is mutated. For this, egg cannibalism by *ppk23* mutant larvae was assayed when eggs were either dechorinated or hexane washed. Indeed, *ppk23* mutant larvae cannibalized eggs from both treatments (Fig 3D). In a subsequent assay, *ppk23* mutant larvae also cannibalized hexane-washed eggs that were perfumed with 7,11-HD (panel A in S5 Fig), thus suggesting that the function of *ppk23* required for hydrocarbon detection is conserved across the developmental stages and, interestingly, modulates different behaviors in larvae and adults. Given that larvae might concurrently sense the wax layer as an aversive cue through other sensory pathways (olfactory or gustatory), we further assayed whether larvae that are mutant for gustatory receptor (*Gr33a*), odorant coreceptor (*Orco*), and ionotropic receptor (*Ir25a*) cannibalized dechorinated eggs. All tested mutants and their respective control (wild-type) larvae abstained from cannibalizing dechorinated eggs but cannibalized eggs treated with hexane (panels B–D in S5 Fig). All in all, these results suggest that the deterrent effect of the wax layer was not mediated by the function of the classical set of chemosensory systems (*Gr*, *Or*, and *Ir*) but is limited to the *ppk*-dependent sensory system. However, since our understanding of how larvae detect their gustatory and pheromonal environment at present is rather limited [55, 56], further work is necessary to identify the *ppk23*-dependent neuronal circuits that respond to 7,11-HD to regulate egg cannibalism.

### Maternal pheromones prevent leakage of egg contents

Earlier studies have reported the water proofing nature of an egg’s wax layer [37] that can be compromised using organic solvents [57] to facilitate eggshell permeability (especially during histological preparations). We therefore next morphologically examined the surface of wax-layer–deprived eggs to understand why they become vulnerable to cannibal larvae and found that they extrude egg contents through their vitelline membranes. Fine fluid droplets appeared on the surface of hexane-treated eggs after about 20–25 min (Fig 3B). However, such droplets were not present on dechorinated or intact eggs (Fig 3A–3C). Cryo-scanning electron microscopy of the egg surface confirmed the droplets to be egg contents permeating through the vitelline membrane (Fig 3C; panels A–D in S7 Fig). Interestingly, in most eggs, these droplets stabilize (i.e., they do not completely drain the egg) and persist as such for several hours. This suggests that eggs might have repair mechanisms or that the extruded material could be blocking the permeating sites.

To further ascertain the extent to which maternal and paternal pheromones in the wax layer contribute towards leak-proofing an egg, we examined dechorinated eggs of the four *oe* mutant crosses (described earlier) for leakage under both light and electron microscopy. We found that only eggs with *oe*^−^ mutant motherhood that had reduced female pheromones were leaky, suggesting the involvement of maternal pheromones present in the wax layer in preventing egg leakage (panel A in S6 Fig; panels E–H in S7 Fig). However, when hexane-washed eggs perfumed with 7-T, cVA, 7,11-HD, and 7,11-ND were examined for leakage using a similar setup, we found that all eggs leaked irrespective of the added pheromone (panel B in S6 Fig). We nevertheless observed some differences among the eggs perfumed by the four hydrocarbons in a) the extent to which they leaked and b) the persistence of the leak. However, we were unable to quantify these differences empirically, and thus, despite demonstrating a strong link between maternally provisioned hydrocarbons and egg leakage, this assay could not independently tag 7,11-HD to the leak-proofing mechanism. This could also be possibly attributed to the arbitrary concentration of 7,11-HD we use or because perfuming fails to completely remodel the wax-layer structure artificially in the absence of other synergetic pheromones.

### Leaked egg content provides gustatory cues

The above finding that altering the wax-layer composition through either chemical treatment or genetic manipulation leads to extrusion of egg contents through the vitelline membrane led us to test whether this leaking egg content makes eggs vulnerable to cannibals. We first assayed larval movement in the presence of intact and injured eggs in agar-lined Petri plates to quantify changes in foraging patterns. However, unlike cannibalistic aggregation occurring around injured larva that we have previously reported [26], groups of injured eggs did not elicit larval aggregation, and the injured eggs were only cannibalized when they were accidentally encountered (Fig 3E). However, 30% of the generally invulnerable dechorinated eggs (Fig 1F) succumbed to cannibals when smeared with egg content leaking from injured eggs (Fig 3F). These results imply that larval recognition and cannibalism of conspecific eggs relies on specific gustatory cues emanating from leaking egg content. Nevertheless, this does not rule out the possibility that leaky or injured eggs might additionally release other stress/injury responsive signals that act as cues, facilitating larval detection.

### 7,11-HD layer can mask sensory cues

Since several experiments above suggested that the protective role of 7,11-HD is dose independent (Figs 1F, 2C and 3F), we speculated that the wax layer in general and 7,11-HD in particular form a physical layer that masks or conceals the egg’s nutrient content. To validate our hypothesis, we tested whether drops of yeast that are attractive to larvae lose their ability to do so when coated externally with a layer of 7,11-HD. We thus assayed larval detection and feeding of such 7,11-HD–coated yeast droplets that were dyed to score larval feeding. Larval feeding on yeast coated with 7,11-HD was much lower than in the control treatment in which the yeast was coated with the solvent hexane (Fig 3G). Interestingly, when the 7,11-HD layer on the yeast was damaged with a needle (analogous to injuring an egg), larval feeding was no longer reduced despite the presence of 7,11-HD (Fig 3G). *ppk23* mutant larvae, in contrast, showed no reduction of larval feeding on yeast droplets coated with 7,11-HD (Fig 3G). These results suggest that 7,11-HD forms a physical layer that prevents emanation of cues from substances (yeast or egg) it envelops. However, damage to this layer compromises this effect, even though the same quantity of 7,11-HD remains on the surface.

### Chemical deception might deter egg cannibalism

Our study shows that under laboratory conditions, the wax layer in *D*. *melanogaster* eggshell a) protects eggs from cannibal larvae, b) prevents egg contents from leaking out through the vitelline membrane, and c) contains pheromones provisioned by both parents. Together with the already-reported result that ovipositing females mark egg-laying sites with a bouquet of pheromones [51], our finding suggests a nonvisual deception mechanism, whereby larvae misclassify conspecific eggs as an inedible object. This deception seems to be mediated by the hydrocarbon-laden wax layer that conceals the embryo and consequently silences the cues that reveal its identity. However, when damaged or leaky, changes in tactile and gustatory cues might facilitate larval detection of these eggs.

We speculate below on several possible mechanisms through which such a nonvisual deceptive egg defense could possibly operate: first, through “chemical insignificance” [58], whereby cannibals may not detect or recognize an egg owing to a lack of a detectable chemical profile; second, through “chemical masquerade” [59] whereby, despite detection, the cannibals might misclassify eggs as objects they normally ignore, possibly empty eggshells. Third, since *D*. *melanogaster* mark oviposition sites with a similar bouquet of hydrocarbons [51], the eggs might blend with the chemical profile of the environment, additionally preventing detection through “background matching” [60]. However, since eggs remained protected on the pheromone-free agar surfaces we used during our assays, background matching may be less crucial. Nonetheless, as female aggregation at these oviposition sites increases over time, the cumulative on-site pheromone concentrations might increase to extents that could provide enhanced background matching for eggs that are laid later. Thus, communal egg-laying behavior in *D*. *melanogaster* may facilitate chemical camouflage through impregnation of maternal pheromones onto the egg, a trait that is likely to be favored by natural selection. Interestingly, since oviposition sites are generally shared among several sibling *Drosophila* species [27], such chemical protection of eggs may have extended effects on intraspecific predation and calls for further investigation [49].

Another potential and inclusive possibility is that the wax layer merely serves as a protective layer to the egg with a primary role in preventing desiccation and, as a byproduct, acts as a preventive barrier against cannibals, pathogens, and toxin permeability. This view is also supported by the parsimonious nature of arthropod pheromones [33], which allows us to safely speculate that the pheromones in the wax layer of fly eggs could subserve other functions in diverse contexts [41]. For example, the pheromones in the wax layer could serve as conspecific cues for adult aggregation, modulate the number of eggs laid at a given site, act as a dispersal cue, and possibly mediate kin recognition. More broadly, our results empirically demonstrate the multiple independent context-dependent functions of a specific pleiotropic pheromone within a single species.

In conclusion, deception as an antipredatory strategy has been predominantly studied in the visual sense but is certainly widespread in other sensory modalities, too [61]. The few studies describing chemical deception show chemical matching but generally do not study the sensory or the mechanism underlying such deception (for example, masquerade, background matching) [62, 63]. Our work here possibly demonstrates chemical deception being mediated by pheromones, which might additionally match an environment created by the ovipositing females. This also mechanistically differs from “chemical mimicry,” for example, in which social parasites mimic host individuals [64]. While studies on animal deception have rarely used model systems, our study demonstrates that doing so opens the way for research on the sensory, neural, genetic, and mechanistic basis of deception at an unprecedented resolution.

## Materials and methods

### *D*. *melanogaster* lines and rearing conditions

All fly lines were reared on standard cornmeal/yeast medium (15 g agar, 30 g sucrose, 60 g glucose, 12.5 g dry yeast, 50 g cornmeal, 0.5 g MgSO_4_, 0.5 g CaCl_2_, 30 mL ethanol, 6 mL propionic acid, and 1 g nipagin per liter of water) at 25°C and 70% relative humidity [26]. Canton S larvae and eggs were used as the wild-type line for most of the behavioral and biochemical assays (Figs 1, 2A, 2E, and Fig 3) unless explicitly mentioned otherwise. The eggs and larvae used for the assays were randomly allocated to different treatments.

*PromE(800)-Gal4 [4M]*,*Tub*:*Gal80ts* flies, *UAS-StingerII*, *UAS-Hid/CyO* flies, and *UAS-StingerII* flies were used to generate crosses for *oe* ablation experiments (Fig 2B and 2C; panel C in S1 and S4 Figs, panel A in S6 Fig, panel E–H in S7 Fig) as described previously [39]. Briefly, apoptosis was induced in adult flies after eclosion, by overnight heat shock at 30°C for four consecutive days. However, little fluorescence was observed in some of these mutant flies even after the heat-shock procedure. *Orco*; *IR25a*; *Gr33a-Gal4*; *UAS-hid*, *rpr*; *w1118*; and BAC-rescue constructs for *Orco* and *Ir25a* were used to assay the influence of smell and taste on larval ability to cannibalize conspecific eggs [65, 66]. *ppk23* mutant larvae were used to assay their response towards 7,11-HD [54].

### Behavioral experiments

#### Larval survival upon hatching (Fig 1A and 1B)

*Drosophila* females were allowed to oviposit on egg-laying medium (orange juice/agar). The eggs were transferred to agar plates, and upon hatching, first-instar larvae were collected and placed individually in a 24-well cell culture plate layered with 2% agar. Four replicate plates were set up, and larval mortality was scored daily until all larvae died. For a follow-up experiment, first-instar larvae were similarly set up in a 24-well cell culture plate individually. They were either maintained as such (unfed) or fed a single meal of two injured eggs. Four replicate plates per treatment were set up, and the number of larvae surviving four days later was recorded.

#### Egg vulnerability (Figs 1F, 2C and 2D, 3F and S1 and S5)

Vulnerability of eggs to cannibalism was assayed by placing groups of five eggs (1 h old) in agar vials containing 10 second-instar Canton S larvae starved for 2 h prior to the assay. The number of eggs present in the vial was scored 1 h, 4 h, 8 h, and 12 h after the assay was set up. The few eggs that hatched during the last time point were excluded for analysis. The eggs used for assays were treated as follows: a) intact, washed with water; b) dechorinated, 2 min wash in commercial bleach (5.25% sodium hypochlorite diluted 1:1 with water) and thoroughly rinsed with water; c) hexane washed, dechorinated eggs washed in hexane for 2 min and rinsed with water; and d) injured, intact eggs were pricked with a fine tungsten bristle (0.15-mm thickness). The above egg vulnerability assay was slightly modified for experiments depicted a) in Fig 3E, in which dechorinated eggs were smeared with egg content leaking from injured eggs, and b) in S5 Fig, in which larvae that were mutants for various sensory receptors (*ppk23*, *Gr33a*, *Orco*, *Ir25a*) were tested along with Canton S (wild-type) larvae as control.

#### Egg toxicity (Fig 1C and 1D)

For assaying the toxicity of *Drosophila* eggs, we set up 5 rearing vials containing 10 mL of standard cornmeal/yeast medium laced with 150 μL of egg extract (3,000 eggs homogenized in 1,800 μL of 0.1% saline) and seeded them with 50 eggs. Similar vials laced with 50 μL of saline were set up as controls. The proportion of larvae successfully pupating and the mean larval developmental period were calculated by scoring pupation in each vial daily.

#### Egg perfuming (Fig 2D)

The commercially synthesized pheromones (7-T, cVA, 7,11-HD, and 7,11-ND) from Cayman Chemical (original concentration 5 × 10^5^ μM; Cayman Chemical, Ann Arbor, MI, USA) were serially diluted in hexane. Two μL each of these diluted pheromones were added onto three groups of five hexane-washed eggs placed on glass slides and allowed to air dry for 1 min. The eggs were then transferred using fine brushes into agar vials containing 10 second-instar larvae that had been starved for 2 h, and the number of eggs cannibalized was recorded 8 h later.

#### Egg leakage (Figs 3A and 3B and S6)

Intact, dechorinated, and hexane-washed eggs were placed in Petri plates lined with 2% agar and examined under a stereo microscope for an hour. The eggs were photographed at regular intervals using a Canon 7D DSLR camera mounted on a Leica stereo microscope. A similar setup was used to examine dechorinated eggs of the four *oe* crosses (panel A in S6 Fig). The protocol was slightly modified to examine leakage in hexane-washed eggs that were perfumed with four pheromones (7-T, cVA, 7,11-HD, and 7,11-ND). Hexane-washed eggs were first perfumed with 2 μl of the four pheromones at the highest concentration (5 × 10^5^ μM) on glass slides and then transferred onto agar-lined Petri plates for imaging (panel B in S6 Fig).

#### Larval attraction to injured eggs (Fig 3E)

To quantify larval attraction to injured eggs (potential victims), we presented groups of 20 larvae with a choice between five intact eggs and five eggs that were pricked with a fine tungsten bristle (0.15-mm thickness) in 90-mm Petri plates lined with 2% agar. Second-instar Canton S larvae (92–94 h old, counted from egg laying) were placed at the center of the Petri plate at time 0; the plates were photographed at 15 and 30 min. The proportion of larvae within a 5-mm radius around the intact and injured eggs at each time point was counted from the images.

#### Yeast perfuming (Fig 3G)

To assay whether 7,11-HD effectively masks gustatory and olfactory cues emanating from yeast, we tested whether larvae detect yeast drops that are coated with 7,11-HD. Droplets of yeast paste mixed with indigo carmine dye (0.04 mg/1 g yeast; Sigma Aldrich, St. Louis, MO, USA) was placed on small plastic discs (3 mm) and allowed to dry for 3 h, then three successive layers of 0.5 μM 7,11-HD diluted in hexane were dripped over the yeast and allowed to dry over 10-min intervals. As a control, yeast drops were coated with hexane alone. To further demonstrate that the damaged 7,11-HD layer is inefficient at masking the yeast, we included another treatment in which yeast drops perfumed with 7,11-HD as described above had their surfaces scratched with a fine needle. Individual yeast discs were then placed centrally on a Petri plate (60 mm) lined with agarose (2.5%). Eight replicate plates per treatment were thus set up. Groups of 10 second-instar larvae (Canton S and *ppk23* mutant; starved for 30 min) were then placed onto the Petri plate. After 30 min, the larvae were visually inspected for feeding (presence of dye in their guts) and scored.

### Statistical methods

Most experiments were analyzed using ANOVA in JMP v.10. The proportional data from the following assays were arcsine-square–root transformed and analyzed appropriately using either an ANOVA or a Welch’s *t* test for unequal variance: egg vulnerability, egg toxicity, yeast perfuming, and larval attraction to injured eggs. The data from the egg perfuming assay were analyzed by logistic regression with overdispersion, wherein the four pheromones were included with interaction (PROC GLIMMIX) and pheromone concentration was log transformed and treated as a continuous variable. To simplify the analysis, the three replicates per pheromone concentration were pooled. Statistical analysis of data from mass spectroscopy is described in detail within the respective section below.

### GC-MS and APPI-FT-ICR-MS protocol

#### Hexane extract of the wax layer

70 mg of fertilized eggs (approximately 6,000) were dechorinated, rinsed in water and transferred into a 1-mL glass vial (Sigma Aldrich). 500 μL of n-Hexane (Sigma Aldrich) was added to these eggs and agitated gently on a vortex for 5 min; the solvent was then transferred to fresh vials using glass Pasteur pipettes and assigned codes “blinded” prior to analyzing them.

#### GC-MS

A gas chromatograph (Trace 1300 GC, Thermo Scientific, Bremen, Germany) hyphenated with a quadrupole mass spectrometer (TSQ 8000 Evo, Thermo Scientific) was used for the study. Hydrocarbons were separated on a capillary column (30 m × 0.25 mm I.D. with 0.25 mm film thickness, Zebron ZB-5ms, Phenomenex) using the following temperature program: initial temperature 50°C held for 3 min, ramped to 150°C at 5°C/min, ramped to 300°C at 15°C/min, and held for 2 min. Helium was used as a carrier gas at a constant flow of 1 mL/min. Injections of 2 μL of eggs’ extracts were made in a splitless mode. The injection port and transfer line temperature were kept at 250°C, and the ion source temperature set at 200°C. Ionization was done by electron impact (EI, 70 eV), and acquisition performed in Full Scan mode in the mass range 50–550 *m*/*z* (scan time 0.2 s). Each of the standard solutions of pheromones at 0.1 mg/mL in hexane were injected and imported in the NIST databases of EI mass spectra. Identification of hydrocarbons was done using XCalibur (Thermo Scientific) and NIST 14 library. The total ion current (TIC) MS was integrated, and the percent of peak area (compared to the total peak area) calculated for each of the four pheromones (cVA, 7-T, 7,11-HD, and 7,11-ND) identified.

#### APPI FT-ICR MS

The high-resolution mass spectrometry studies were carried out using a hybrid linear ion trap Fourier transform ion cyclotron resonance mass spectrometer (LTQ FT-ICR MS, Thermo Scientific) equipped with a 10 T superconducting magnet (Oxford Nanoscience, Oxon, UK). The mass spectrometer was equipped with the narrow aperture detection electrodes (2X NADEL) ICR cell (Spectroswiss, Lausanne, Switzerland) containing four detection and four excitation electrodes based on a design of an open-ended cylindrical ICR cell, as described elsewhere [67]. The instrument was operated via standard built-in data station and instrument control software (Xcalibur, Thermo Scientific).

Positive ions were generated using atmospheric pressure photoionization (APPI) ion source, injected into the linear ion trap for accumulation, and further transferred into the 2X NADEL ICR cell through a set of multipole ion guides. APPI source temperature was 300°C, and inlet capillary temperature was 200°C. Ion population inside the linear ion trap was controlled via the automatic gain control (AGC) function (Thermo Scientific). The ions confined inside the 2X NADEL ICR cell via gated trapping were further excited using a dipolar broadband frequency-sweep excitation. Quadrupolar ion detection was used to acquire analyte ion signals in the cyclotron frequency regime, providing high mass accuracy measurements [38]. Ion signal detection period was 768 ms, corresponding to the resolving power of 140,000 at 400 *m*/*z*. Experimental time domain signals (transients) were recorded using the *.dat file format via advanced user interface capabilities (Thermo Scientific). 100 single transients were summed to improve the signal-to-noise ratio (SNR) and identify low abundant peaks. The final transient was zero-filled twice, apodized with the Hann window, and Fourier transformed into a frequency spectrum. The peaks were picked using three-point parabolic interpolation. Signal processing and data visualization were performed using Peak-by-Peak data analysis framework (Spectroswiss) [68].

### Data analysis

All the high-resolution mass spectra were acquired in the mass range of 280–500 *m*/*z* with the AGC value of 5 × 10^5^. The mass spectra were externally calibrated with the calibration mixture containing caffeine, MRFA, and ultramark (Buchs, Switzerland). Further, mass spectra were internally recalibrated using monoisotopic peaks of the three reference compounds (cVA, 7,11-HD, and 7,11-ND; Cayman Chemical) and a standard two-parametric calibration equation.

First, the mass spectrum of hexane extract of wax layer of intact eggs of wild-type flies was analyzed (Fig 2A). The full list of compounds identified in this mass spectrum is shown in the S1 Table. In general, several ion types of each identified compound were detected: M^+^˙, [M+H]^+^, [M+H-H_2_]^+^, [M+H-2H_2_]^+^, [M+H-H_2_+H_2_O]^+^_,_ and [M+H-2H_2_+H_2_O]^+^. The same ion types and corresponding ionization mechanisms using an APPI source have been previously reported [69]. The mass spectra of some ions of interest are shown in S2–S4 Fig. Only the monoisotopic peak (^12^C) of the main ion (the highest abundance) of an identified compound is indicated in S1 Table. Additionally, several target compounds were commercially synthesized (Cayman Chemical), diluted in the hexane with the concentration of 0.5 mg/mL, and MS analyzed under identical experimental conditions (AGC = 1 × 10^5^). The corresponding mass spectra, displaying four major (the highest abundance) ions of commercially synthesized cVA, 7-T, 7,11-HD, and 7,11-ND, demonstrate the same spectral composition (ion types) of the compounds as in the mass spectrum of hexane extract of the wax layer of intact eggs of wild-type flies (S3 Fig).

Further, hydrocarbon profile of the wax layer was investigated on eggs laid by four parental crosses generated from males and females, with (*oe*^+^) or without (*oe*^−^) *oe*s. The intensities of cVA, 7-T, 7,11-HD, and 7,11-ND were calculated from mass spectra of hexane extracts of wax layer of eggs laid by transgenic mutant fly crosses with/without ablated *oe*s (*oe*^−^): C1, *♂oe*^+^ × *♀oe*^+^; C2, *♂o*e^−^ × *♀oe*^−^; C3, *♂oe*^−^ × *♀oe*^+^_;_ and C4, *♂oe*^+^ × *♀oe*^−^. Three mass spectra of each hexane extract (C1–C4) were acquired in separate runs, 12 mass spectra in total. The intensity of each target compound is the summation of the SNRs of the monoisotopic peaks of the four most abundant ions, averaged through the three corresponding mass spectra (Fig 2B and S2 Table). SNR was calculated as follows: SNR = I_peak_/(5 ∙ δ_noise_), where I_peak_ is the absolute spectral intensity of an analyte peak and δ_noise_ is the SD of noise. S4 Fig demonstrates the expanded views of single mass spectra of hexane extracts C1–C4 plotted on the same figure. To correct drift in MS response (number of charges’ variation between measurements), all the mass spectra were normalized by employing the total SNR. The total SNR of a mass spectrum was calculated as sum of intensities of all peaks higher than 5 ∙ δ_noise_. In general, the total SNR variation between measurements was less than ±10%.

### Cryo-scanning electron microscopy

The surfaces of five dechorinated and five hexane-washed eggs of Canton S were examined by cryo-scanning electron microscopy (S7A–S7D Fig). Similarly, five dechorinated eggs of each *oe* mutant cross were also examined by environmental scanning electron microscopy (S7E–S7H Fig). For cryo-scanning electron microscopy, we used a Quorum system PP3010T attached to a Helios 650 (FEI Company, Eindhoven, The Netherlands). The eggs were mounted on aluminum stubs using a mixture of Tissue-Tek (Sakura Finetek Europe, Alphen aan den Rijn, The Netherlands) and colloidal graphite (Agar Scientific, Stansted, Essex, UK), frozen in nitrogen slush at −210°C and then transferred to the preparation chamber of the Quorum system. The sample was freeze-dried at −80°C for 10 min and then sputter coated with platinum at 10 mA for 25 s. After transfer on the cryostage at −140°C in the scanning electron microscope, imaging was performed at 5 keV using an Everhart-Thornley electron detector [70]. Some experiments were done under low-vacuum 300–400 Pa conditions. Fresh eggs were directly mounted on a scanning electron microscopy stub and imaged with the low-vacuum large field detector in the Quanta 250.

## Supporting information

S1 Fig Control bioassays for various experiments. (A, B) Effect of removing egg layers on egg development: (A) egg hatching success (mean ± SE), (B) egg developmental period (mean ± SE), and (C) egg-to-adult viability (mean ± SE) of *D*. *melanogaster* eggs that were intact, dechorinated, and hexane washed after dechorination (*n* = 5 replicate vials). Hexane treatment did not affect hatchability (ANOVA: *F*_*1*,*8*_ = 0.058, *p* = 0.82), slightly shortened the egg developmental period (ANOVA: *F*_*1*,*8*_ = 5.21, *p* = 0.0519), and reduced egg-to-adult viability marginally. (D) Cannibalism of eggs from unrelated strain (Valais): proportion of eggs from Canton S and Valais strains cannibalized by second-instar Canton S larvae when intact, injured, or hexane washed (*n* = 10 replicate vials). Larvae cannibalized all injured eggs in both strains but did not feed on intact eggs; however, proportion of hexane-washed eggs from both strains were consumed to an identical level. (E) Proportion of adults emerging (mean ± SE) from eggs laid by transgenic mutant (*oe*^−^) flies with ablated *oe*s that were either self-crossed or crossed with wild-type (*oe*^+^) flies (*n* = 4 replicate vials). The wild type (*oe*^+^) had higher viability than the other crosses (ANOVA: *F*_*3*,*12*_ = 4.09, *p* = 0.0324). *p < 0.05, †*P* < 0.1, NS = not significant. Data underlying this figure can be found in S2 Data. NS, not significant; *oe*, oenocyte
(TIF)

S2 Fig High-resolution 10 T APPI FT-ICR MS. Mass spectra of the wax-layer hexane extract of *D*. *melanogaster* eggs (A, G, M) and the hexane control solution (D, J, P). The expanded views of mass regions demonstrate the presence of (B) 11Z,11-octadecen-1-ol-acetate (C_20_H_38_O_2_) and (C) 7Z,11Z-tricosadiene (C_23_H_44_) compounds in the hexane extract and their absence in the hexane solution (E, F); the presence of (H) 7Z,11Z-pentacosadiene (C_25_H_48_) and (I) 7Z,11Z-heptacosadiene (C_27_H_52_) compounds in the hexane extract, and their absence in the hexane solution (K, L); and the presence of (N) 7,11Z-nonacosadiene (C_29_H_56_) and (O) spiro[13,14]octacosane-15-one (C_28_H_52_O) in the hexane extract, and their absence in the hexane solution (Q, R). APPI FT-ICR MS, high-resolution mass spectrometry
(TIF)

S3 Fig Pheromone profile of intact and dechorinated eggs and repeated hexane washes of dechorinated eggs. (A) GC-MS profile of hexane extract of intact (S1) and dechorinated (S2) eggs indicating the peaks corresponding to the four major hydrocarbons (cVA; 7-T; 7,11-HD, and 7,11-ND). (Inset) the relative amount of these hydrocarbons in hexane extract of intact (blue) and dechorinated (red) eggs. (B) Ratio of SNRs of four target pheromones (7,11-HD, 7,11-ND, 7,11-pentacosadiene, 7-pentacosene) to internal calibrants (hexacosane and triacontane) as a function of their concentration ratio in three successive hexane washes of dechorinated eggs. Data underlying this figure can be found in S2 Data. cVa, 11-*cis*-vaccenyl acetate; GC-MS, gas chromatography hyphenated with mass spectrometry; SNR, signal-to-noise ratio; 7-T, 7-tricoscene; 7,11-HD, 7,11-heptacosadiene; 7,11-ND, 7,11-nonacosadiene
(TIF)

S4 Fig High-resolution 10 T APPI FT-ICR MS of commercially synthesized hydrocarbons and eggs laid by *oe* mutants. (A) Mass spectra of commercially synthesized hydrocarbons diluted in hexane. The mass spectra show four major (the highest abundance) ions of 11Z,11-octadecen-1-ol-acetate (cVA; C_20_H_38_O_2_); 7Z-tricosene (C_23_H_48_); 7,11Z-heptacosadiene (C_27_H_52_); and 7,11Z-nonacosadiene (C_29_H_56_). (B) Hydrocarbon profile of the wax layer in eggs laid by four parental crosses generated from males and females, with (*oe*^+^) or without (*oe*^−^) *oe*s. Expanded views of broadband mass spectra of wax layer of transgenic mutant flies with ablated *oe*s (*oe*^−^): cyan, *♂oe*^−^ × *♀oe*^−^; green, *♂oe*^+^ × *♀oe*^−^; blue, *♂oe*^+^ × *♀oe*^+^; red, *♂oe*^−^ × *♀oe*^*+*^; and black, hexane. The views display the intensity (SNR) of monoisotopic peak corresponding to cVA, 7-T, 7,11-HD, and 7,11-ND in the corresponding single mass spectrum. APPI FT-ICR MS, high-resolution mass spectrometry; cVa, 11-*cis*-vaccenyl acetate; *oe*, oenocyte; SNR, signal-to-noise ratio; 7-T, 7-tricoscene; 7,11-HD, 7,11-heptacosadiene; 7,11-ND, 7,11-nonacosadiene
(TIF)

S5 Fig Role of larval chemosensory receptors in sensing 7,11-HD. (A) Proportion (mean ± SE) of hexane-washed (white bars) eggs perfumed with 7,11-HD that were cannibalized by Canton S and *ppk23* mutant larvae (ANOVA: *n* = 4 replicates, 10 eggs/replicate). (B) Proportion (mean ± SE) of dechorinated (dark bars) and hexane-washed (light bars) eggs cannibalized by larvae that lack *Gr33a-Gal4*–positive neurons (*Gr33a-Gal4*/*UAS-hid*,*rpr*) and two control groups (*Gr33a-Gal4*/+ and *UAS-hid*,*rpr*/+) (ANOVA; *n* = 4 replicates, 10 eggs/replicate). (C) Proportion (mean ± SE) of dechorinated (dark bars) and hexane-washed (light bars) eggs cannibalized by *Orco* mutant larvae and its BAC-rescue construct (ANOVA; *n* = 4 replicates, 10 eggs/replicate). (D) Proportion (mean ± SE) of dechorinated (dark bars) and hexane-washed (light bars) eggs cannibalized by *IR25a* mutant larvae and its BAC-rescue construct (ANOVA; *n* = 4 replicates, 10 eggs/replicate). Except for *ppk23* mutant larvae (in Fig 3D), none of the other strains cannibalized dechorinated eggs. However, hexane-washed eggs were cannibalized by all strains to a similar extent. Data underlying this figure can be found in S2 Data. *Grr33a*, gustatory receptor; *Ir25a*, ionotropic receptor; NS, not significant; *Orco*, odorant coreceptor; *ppk23*, pickpocket 23; 7,11-HD, 7,11-heptacosadiene
(TIF)

S6 Fig Egg leakage in eggs laid by *oe* mutants and hexane-washed eggs perfumed with individual pheromones. (A) Pictorial representation of the role of maternal hydrocarbons present within the egg shell in preventing leakage of egg contents. Labels across each row represent eggs laid by transgenic mutant flies with ablated *oe*s (*oe*^−^) that were either self-crossed or crossed with wild-type flies (*oe*^+^). Adjacent bar graph represents the proportion of eggs (mean ± SE) that were found leaking (*n* = 20 eggs per cross) in the corresponding crosses. Leakage of egg content was only observed in eggs transgenically deprived of female hydrocarbons (rows 3 and 4). (B) Pictorial confirmation of the role of four individual pheromones in preventing leakage of egg contents. Labels across each row represent the pheromone with which hexane-washed eggs were perfumed, while the adjacent bar graph represents the proportion of eggs (mean ± SE) that were found leaking (*n* = 20 eggs per treatment) in the respective treatments. Leakage of egg content was only observed in all eggs irrespective of the pheromone they were perfumed with. *oe*, oenocyte
(TIF)

S7 Fig Cryo- and low-vacuum scanning electron imaging of egg leakage. (A–D) Cryo-electron scanning images of hexane-washed egg surfaces sequentially depicting leakage of egg contents through the vitelline membrane taken on Helios. The vitelline membrane distends at weak spots (A), the egg contents then gradually permeate through the distended membrane forming small droplets (B, C), and eventually these droplets merge to form larger droplets (D). (E–H) Low-vacuum electron scanning images (taken on Quanta at low vacuum) of dechorinated eggs laid by transgenic mutant flies with ablated *oe*s (*oe*^−^) that were either self-crossed or crossed with wild-type flies (*oe*^+^): ♂*oe*^+^ × ♀*oe*^+^ (E), ♂*oe*^−^ × ♀*oe*^+^ (F), *oe*^+^ × ♀*oe*^−^ (G), and ♂*oe*^−^ × ♀*oe*^−^ (H). Egg leakage was only observed in *oe*^−^ mutant motherhood eggs (arrowheads in G and H); furthermore, these eggs collapsed under reduced pressure more rapidly than eggs with *oe*^+^ motherhood (E and F). Scale bar in panels (A–D) = 5 μm, and in panels (E–H) = 100 μm. *oe*, oenocyte
(TIF)

S1 Table High-resolution 10 T APPI FT-ICR MS. The compounds (alkenes and alkadienes) were identified in mass spectrum of wax layer’s hexane extract of intact eggs of wild-type flies. Only the monoisotopic peak (^12^C) of the highest abundance ion of an identified compound is indicated in the table. APPI FT-ICR MS, atmospheric pressure photoionization Fourier transform ion cyclotron resonance mass spectrometry
(DOC)

S2 Table High-resolution 10 T APPI FT-ICR MS of hexane extract of wax layer of eggs laid by transgenic mutant fly crosses with/without ablated *oe*s. C1, *♂oe*^+^ × *♀oe*^+^; C2, *♂oe*^−^ × *♀oe*^−^; C3, *♂oe*^−^ × *♀oe*^+^; and C4, *♂oe*^+^ × *♀oe*^−^. The table shows the intensities (SNRs) of four major (the highest abundance) peaks (ion types) of the target compound identified in the corresponding mass spectra. Intensities were obtained from averaging of three mass spectra of each hexane extract acquired with separate runs. The sum of intensities of four major peaks of each compound were used to plot the histogram (Fig 2B). APPI FT-ICR MS, atmospheric pressure photoionization Fourier transform ion cyclotron resonance mass spectrometry; *oe*, oenocyte; SNR, signal-to-noise ratio
(DOC)

S1 Data Raw data for Figs 1, 2 and 3. (XLSX)

S2 Data Raw data for figures in supplementary information. (XLSX)

## Abbreviations

AGC: automatic gain control
APPI FT-ICR MS: atmospheric pressure photoionization Fourier transform ion cyclotron resonance mass spectrometry
a.u.: arbitrary unit; cVA, 11-*cis*-vaccenyl acetate
EI: electron impact
GC-MS: gas chromatography hyphenated with mass spectrometry
*Gr33a*: gustatory receptor
*Ir25a*: ionotropic receptor
LTQ FT-ICR MS: linear ion trap Fourier transform ion cyclotron resonance mass spectrometer
*oe*: oenocyte
*Orco*: odorant coreceptor
*ppk23*: pickpocket 23
RNA-seq: RNA-sequencing
SNR: signal-to-noise ratio
TIC: total ion current
7-T: 7-tricoscene
7,11-HD: 7,11-heptacosadiene
7,11-ND: 7,11-nonacosadiene
