# Supplementary figures and images for: Drosophila melanogaster cloak their eggs with pheromones, which prevents cannibalism
Source: PLoS Biol. 2019 Jan 10;17(1):e2006012. doi: 10.1371/journal.pbio.2006012 (PMC6328083; doi:10.1371/journal.pbio.2006012)

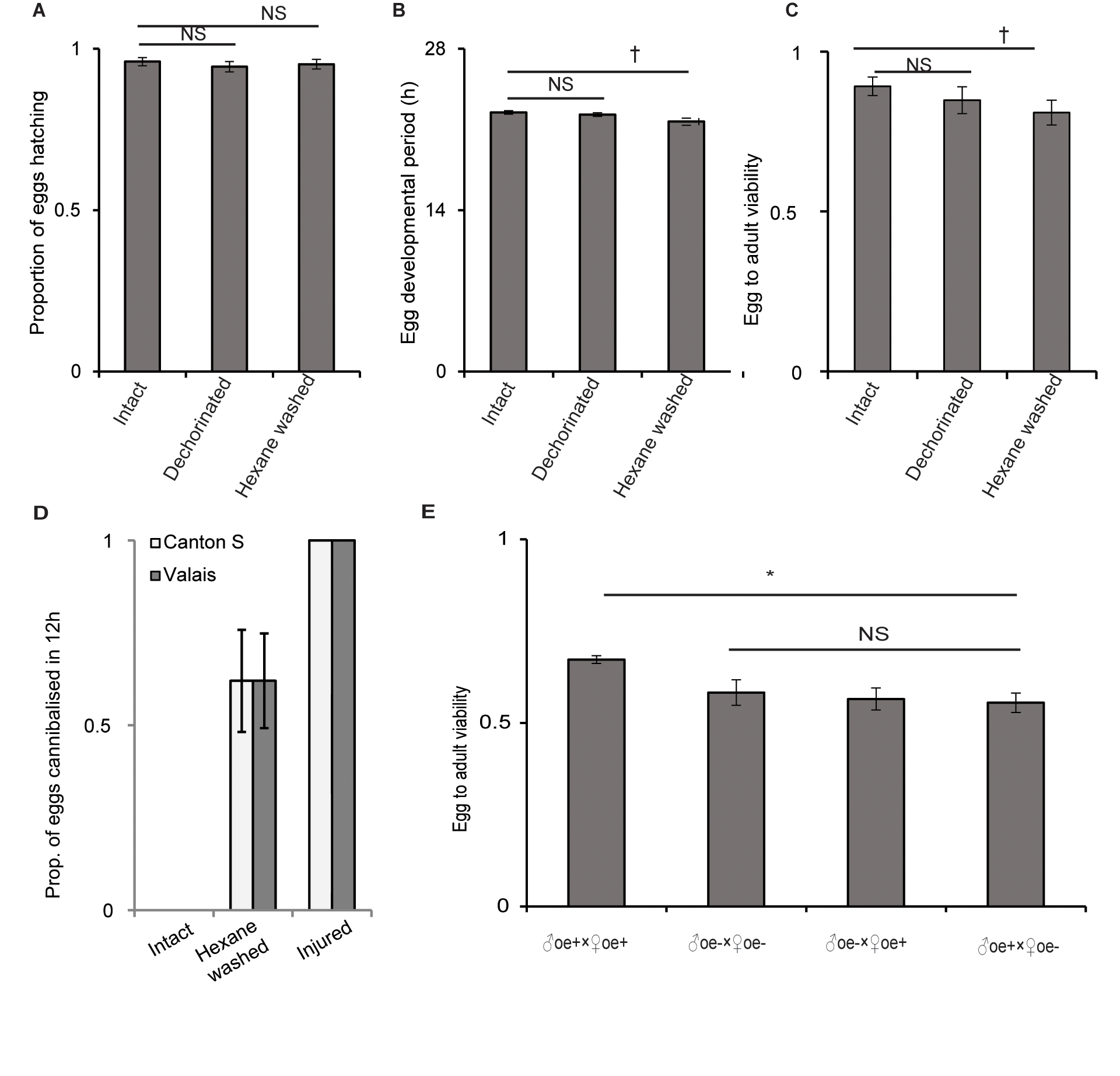

Supplement: S1 Fig — (A, B) Effect of removing egg layers on egg development: (A) egg hatching success (mean ± SE), (B) egg developmental period (mean ± SE), and (C) egg-to-adult viability (mean ± SE) of D. melanogaster eggs that were intact, dechorinated, and hexane washed after dechorination (n = 5 replicate vials). Hexane treatment did not affect hatchability (ANOVA: F1,8 = 0.058, p = 0.82), slightly shortened the egg developmental period (ANOVA: F1,8 = 5.21, p = 0.0519), and reduced egg-to-adult viability marginally. (D) Cannibalism of eggs from unrelated strain (Valais): proportion of eggs from Canton S and Valais strains cannibalized by second-instar Canton S larvae when intact, injured, or hexane washed (n = 10 replicate vials). Larvae cannibalized all injured eggs in both strains but did not feed on intact eggs; however, proportion of hexane-washed eggs from both strains were consumed to an identical level. (E) Proportion of adults emerging (mean ± SE) from eggs laid by transgenic mutant (oe−) flies with ablated oes that were either self-crossed or crossed with wild-type (oe+) flies (n = 4 replicate vials). The wild type (oe+) had higher viability than the other crosses (ANOVA: F3,12 = 4.09, p = 0.0324). *p < 0.05, †P < 0.1, NS = not significant. Data underlying this figure can be found in S2 Data. NS, not significant; oe, oenocyte (TIF) [file pbio.2006012.s001.tif]

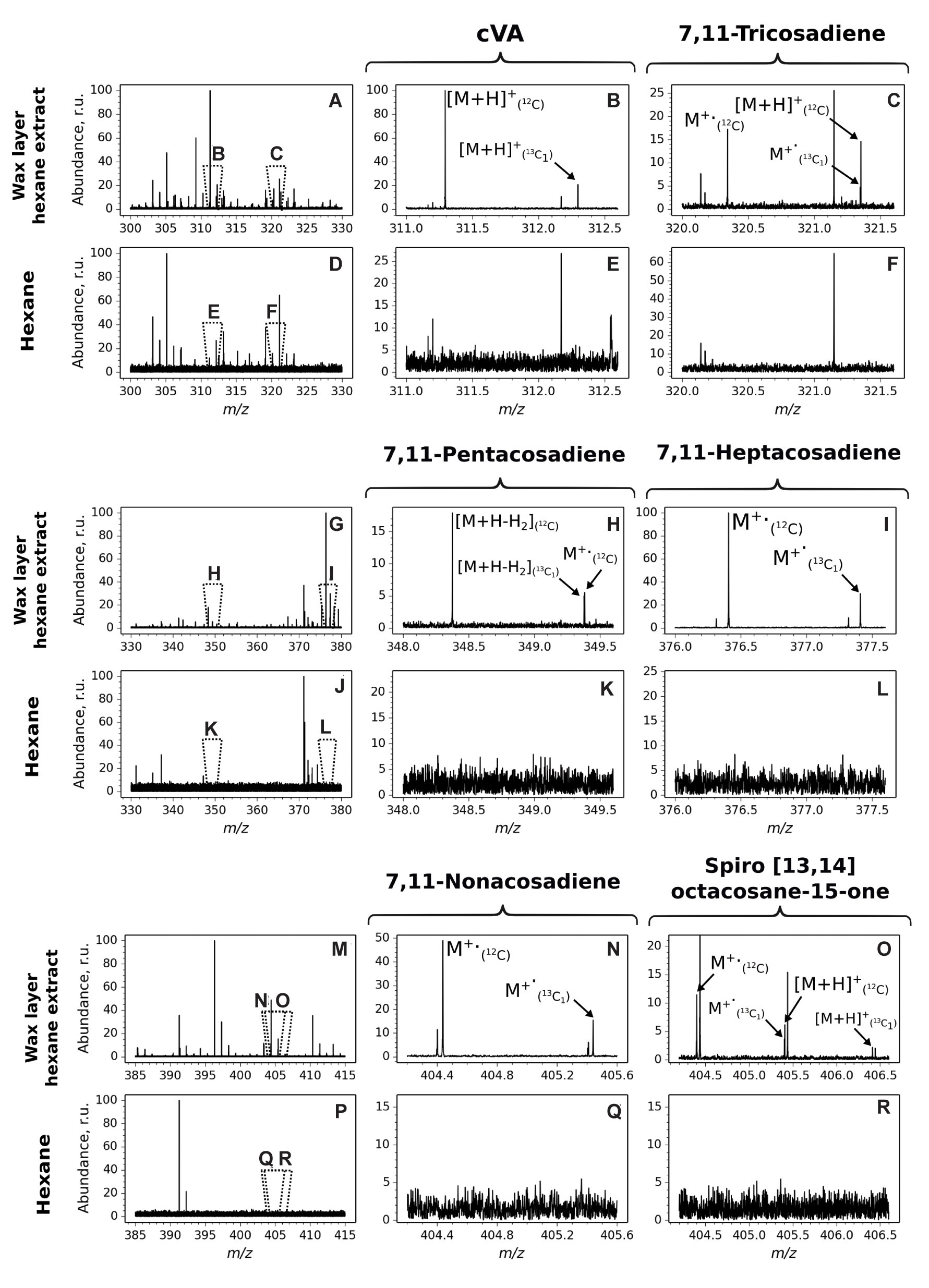

Supplement: S2 Fig — Mass spectra of the wax-layer hexane extract of D. melanogaster eggs (A, G, M) and the hexane control solution (D, J, P). The expanded views of mass regions demonstrate the presence of (B) 11Z,11-octadecen-1-ol-acetate (C20H38O2) and (C) 7Z,11Z-tricosadiene (C23H44) compounds in the hexane extract and their absence in the hexane solution (E, F); the presence of (H) 7Z,11Z-pentacosadiene (C25H48) and (I) 7Z,11Z-heptacosadiene (C27H52) compounds in the hexane extract, and their absence in the hexane solution (K, L); and the presence of (N) 7,11Z-nonacosadiene (C29H56) and (O) spiro[13,14]octacosane-15-one (C28H52O) in the hexane extract, and their absence in the hexane solution (Q, R). APPI FT-ICR MS, high-resolution mass spectrometry (TIF) [file pbio.2006012.s002.tif]

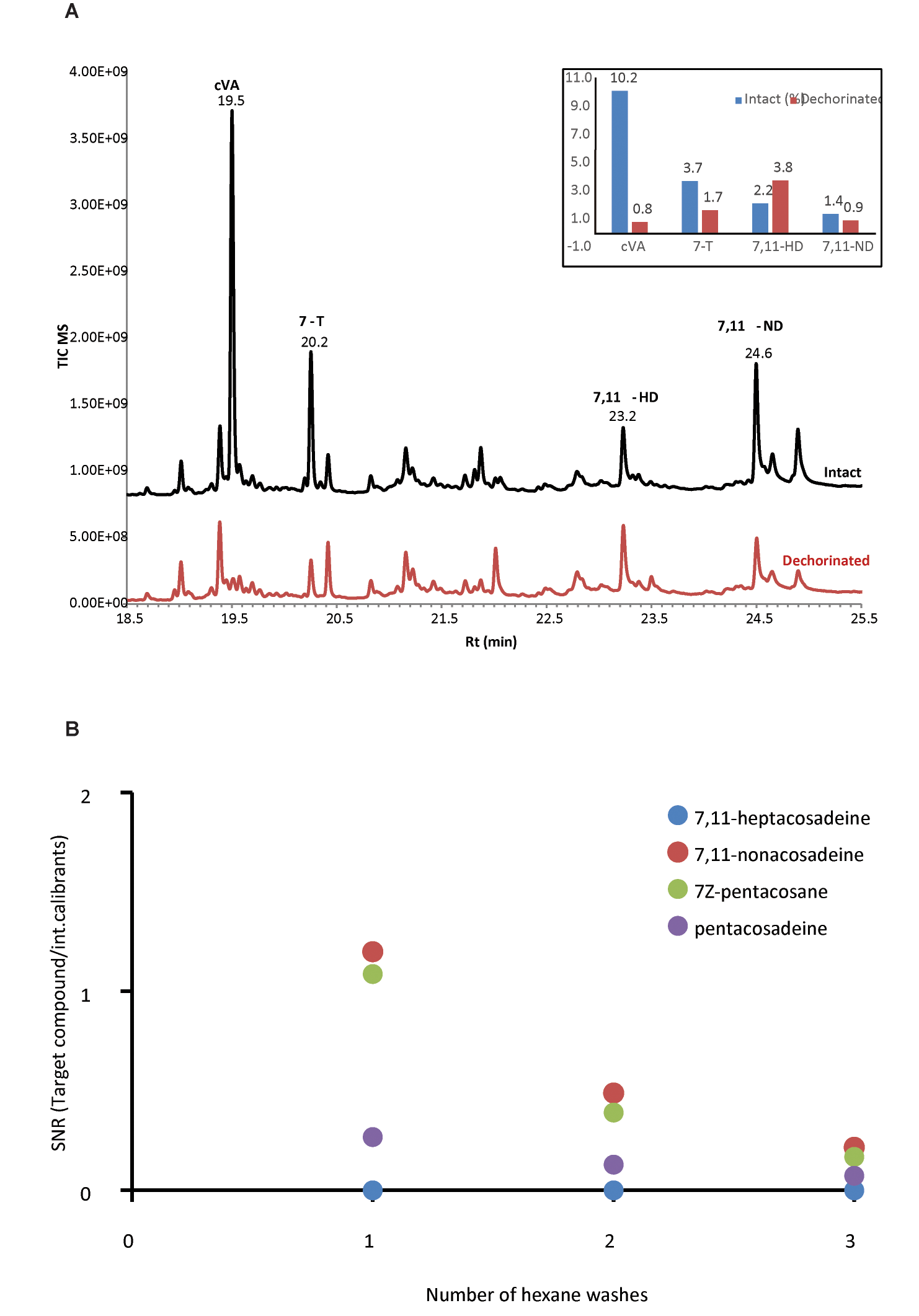

Supplement: S3 Fig — (A) GC-MS profile of hexane extract of intact (S1) and dechorinated (S2) eggs indicating the peaks corresponding to the four major hydrocarbons (cVA; 7-T; 7,11-HD, and 7,11-ND). (Inset) the relative amount of these hydrocarbons in hexane extract of intact (blue) and dechorinated (red) eggs. (B) Ratio of SNRs of four target pheromones (7,11-HD, 7,11-ND, 7,11-pentacosadiene, 7-pentacosene) to internal calibrants (hexacosane and triacontane) as a function of their concentration ratio in three successive hexane washes of dechorinated eggs. Data underlying this figure can be found in S2 Data. cVa, 11-cis-vaccenyl acetate; GC-MS, gas chromatography hyphenated with mass spectrometry; SNR, signal-to-noise ratio; 7-T, 7-tricoscene; 7,11-HD, 7,11-heptacosadiene; 7,11-ND, 7,11-nonacosadiene (TIF) [file pbio.2006012.s003.tif]

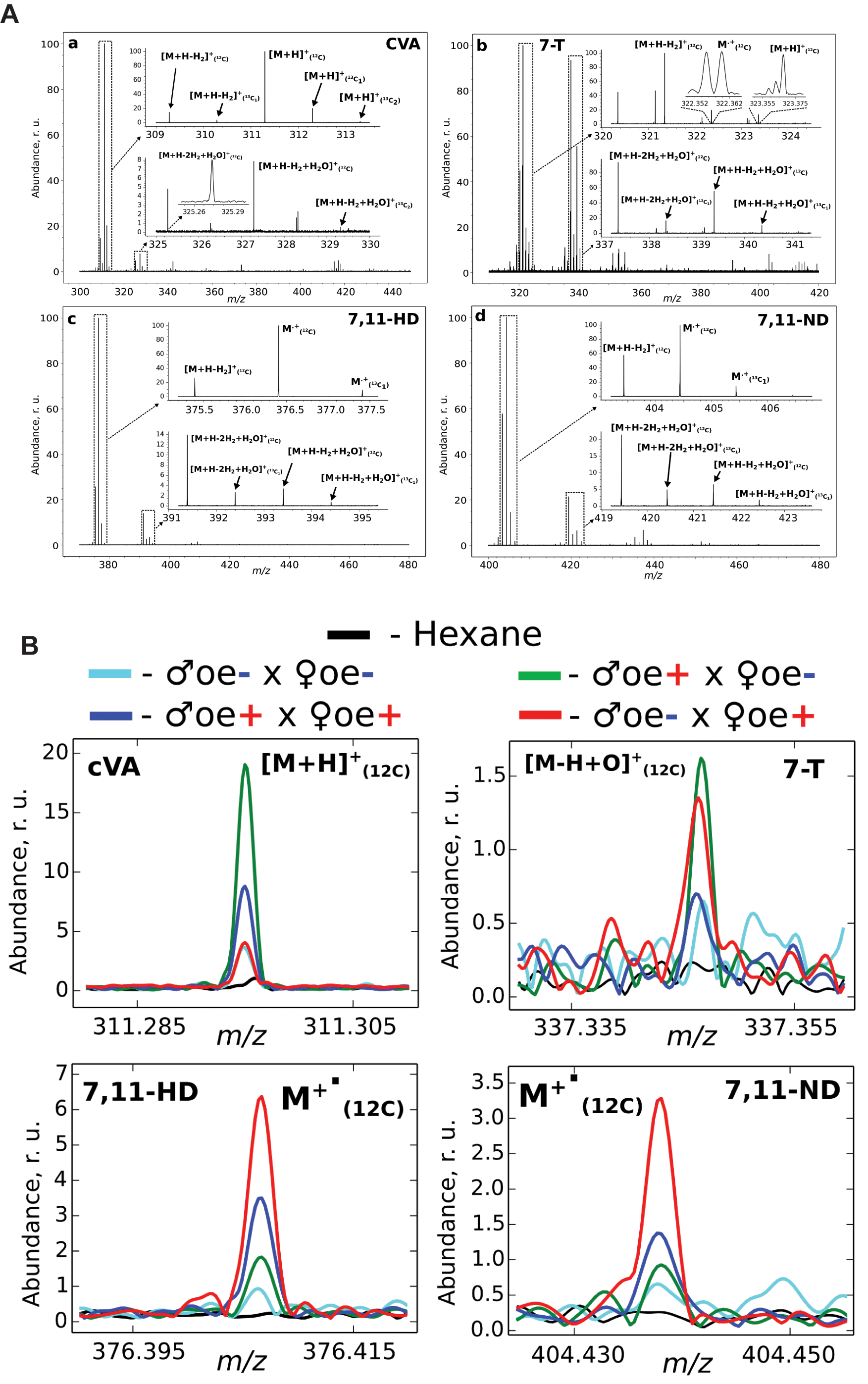

Supplement: S4 Fig — (A) Mass spectra of commercially synthesized hydrocarbons diluted in hexane. The mass spectra show four major (the highest abundance) ions of 11Z,11-octadecen-1-ol-acetate (cVA; C20H38O2); 7Z-tricosene (C23H48); 7,11Z-heptacosadiene (C27H52); and 7,11Z-nonacosadiene (C29H56). (B) Hydrocarbon profile of the wax layer in eggs laid by four parental crosses generated from males and females, with (oe+) or without (oe−) oes. Expanded views of broadband mass spectra of wax layer of transgenic mutant flies with ablated oes (oe−): cyan, ♂oe− × ♀oe−; green, ♂oe+ × ♀oe−; blue, ♂oe+ × ♀oe+; red, ♂oe− × ♀oe+; and black, hexane. The views display the intensity (SNR) of monoisotopic peak corresponding to cVA, 7-T, 7,11-HD, and 7,11-ND in the corresponding single mass spectrum. APPI FT-ICR MS, high-resolution mass spectrometry; cVa, 11-cis-vaccenyl acetate; oe, oenocyte; SNR, signal-to-noise ratio; 7-T, 7-tricoscene; 7,11-HD, 7,11-heptacosadiene; 7,11-ND, 7,11-nonacosadiene (TIF) [file pbio.2006012.s004.tif]

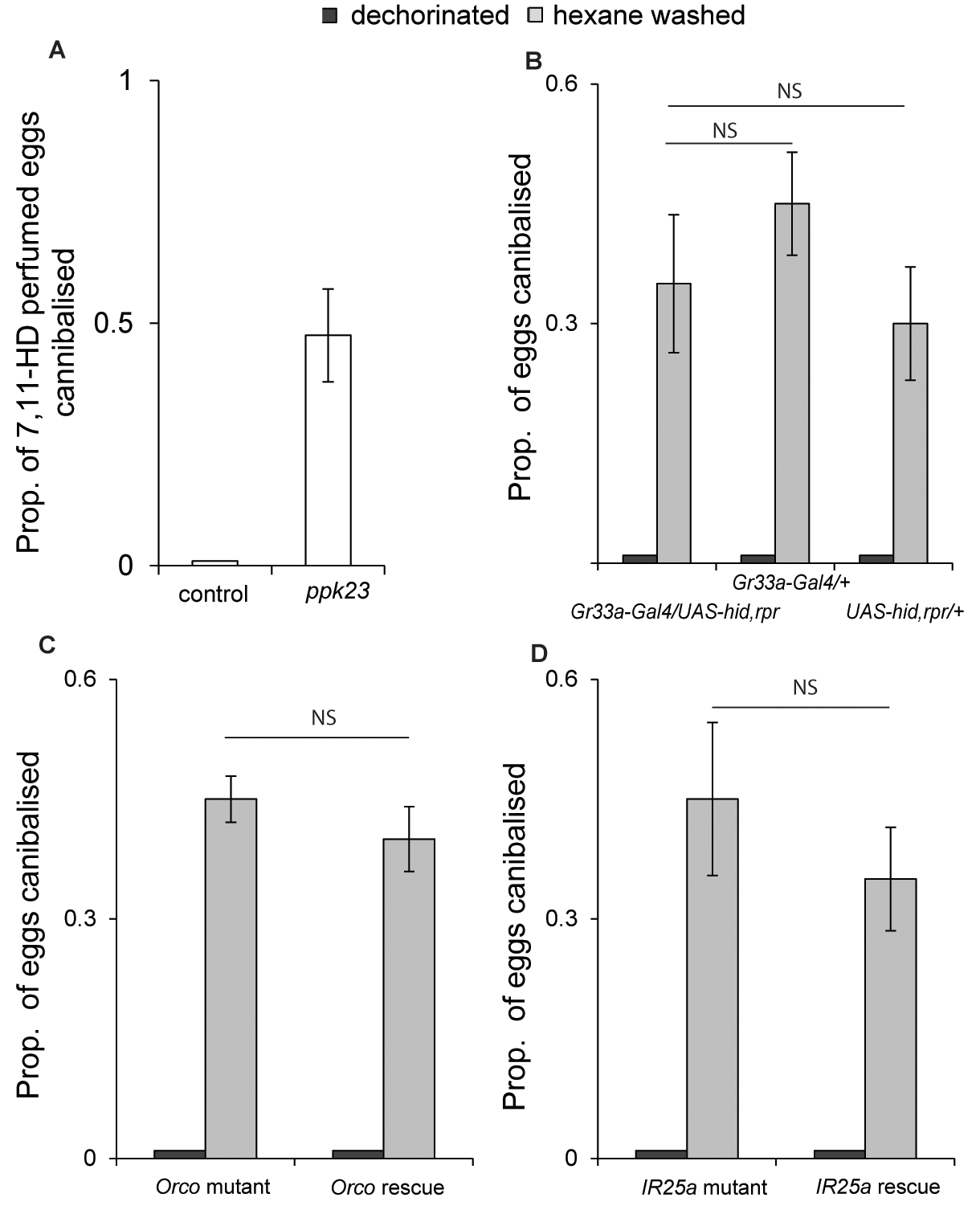

Supplement: S5 Fig — (A) Proportion (mean ± SE) of hexane-washed (white bars) eggs perfumed with 7,11-HD that were cannibalized by Canton S and ppk23 mutant larvae (ANOVA: n = 4 replicates, 10 eggs/replicate). (B) Proportion (mean ± SE) of dechorinated (dark bars) and hexane-washed (light bars) eggs cannibalized by larvae that lack Gr33a-Gal4–positive neurons (Gr33a-Gal4/UAS-hid,rpr) and two control groups (Gr33a-Gal4/+ and UAS-hid,rpr/+) (ANOVA; n = 4 replicates, 10 eggs/replicate). (C) Proportion (mean ± SE) of dechorinated (dark bars) and hexane-washed (light bars) eggs cannibalized by Orco mutant larvae and its BAC-rescue construct (ANOVA; n = 4 replicates, 10 eggs/replicate). (D) Proportion (mean ± SE) of dechorinated (dark bars) and hexane-washed (light bars) eggs cannibalized by IR25a mutant larvae and its BAC-rescue construct (ANOVA; n = 4 replicates, 10 eggs/replicate). Except for ppk23 mutant larvae (in Fig 3D), none of the other strains cannibalized dechorinated eggs. However, hexane-washed eggs were cannibalized by all strains to a similar extent. Data underlying this figure can be found in S2 Data. Grr33a, gustatory receptor; Ir25a, ionotropic receptor; NS, not significant; Orco, odorant coreceptor; ppk23, pickpocket 23; 7,11-HD, 7,11-heptacosadiene (TIF) [file pbio.2006012.s005.tif]

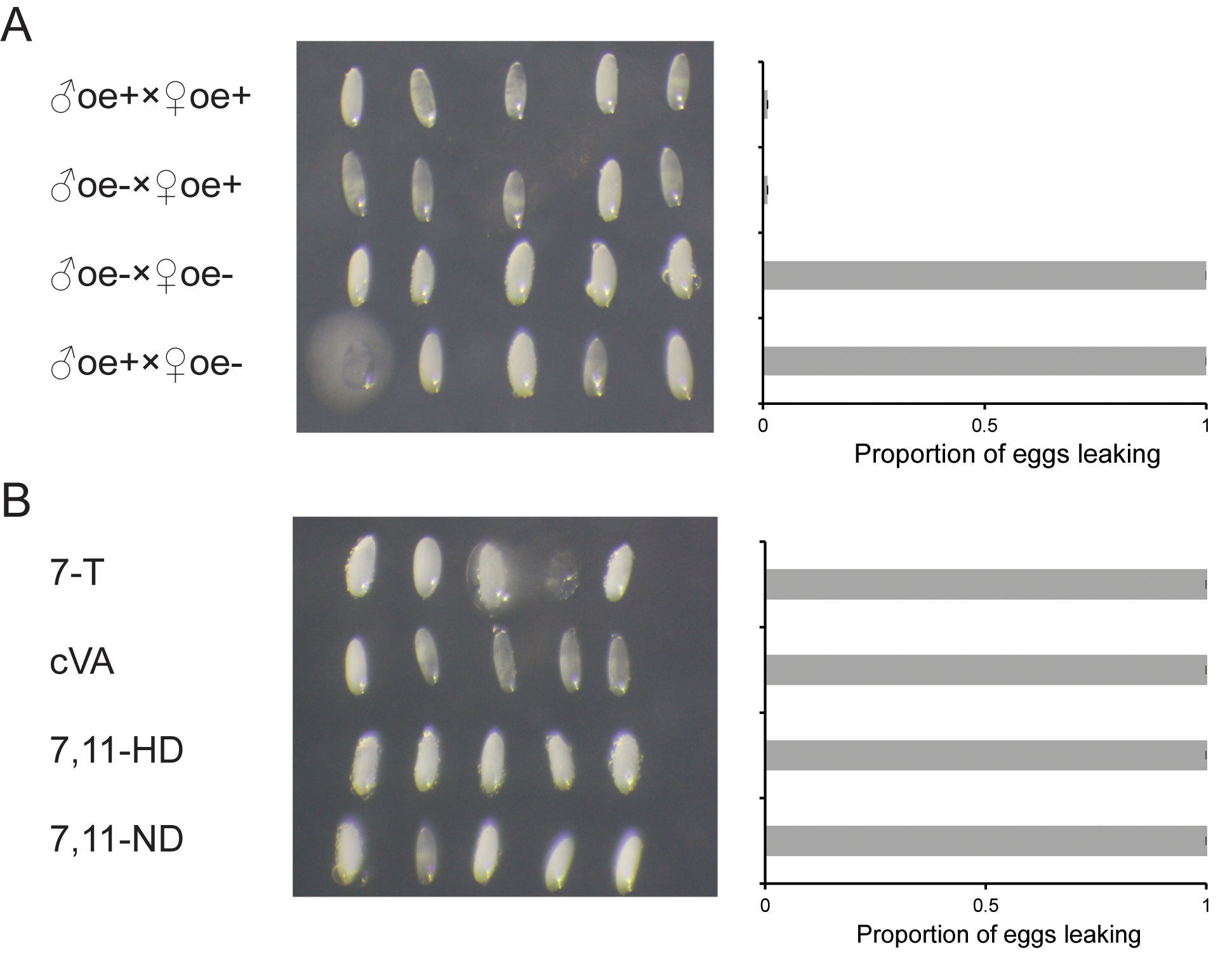

Supplement: S6 Fig — (A) Pictorial representation of the role of maternal hydrocarbons present within the egg shell in preventing leakage of egg contents. Labels across each row represent eggs laid by transgenic mutant flies with ablated oes (oe−) that were either self-crossed or crossed with wild-type flies (oe+). Adjacent bar graph represents the proportion of eggs (mean ± SE) that were found leaking (n = 20 eggs per cross) in the corresponding crosses. Leakage of egg content was only observed in eggs transgenically deprived of female hydrocarbons (rows 3 and 4). (B) Pictorial confirmation of the role of four individual pheromones in preventing leakage of egg contents. Labels across each row represent the pheromone with which hexane-washed eggs were perfumed, while the adjacent bar graph represents the proportion of eggs (mean ± SE) that were found leaking (n = 20 eggs per treatment) in the respective treatments. Leakage of egg content was only observed in all eggs irrespective of the pheromone they were perfumed with. oe, oenocyte (TIF) [file pbio.2006012.s006.tif]

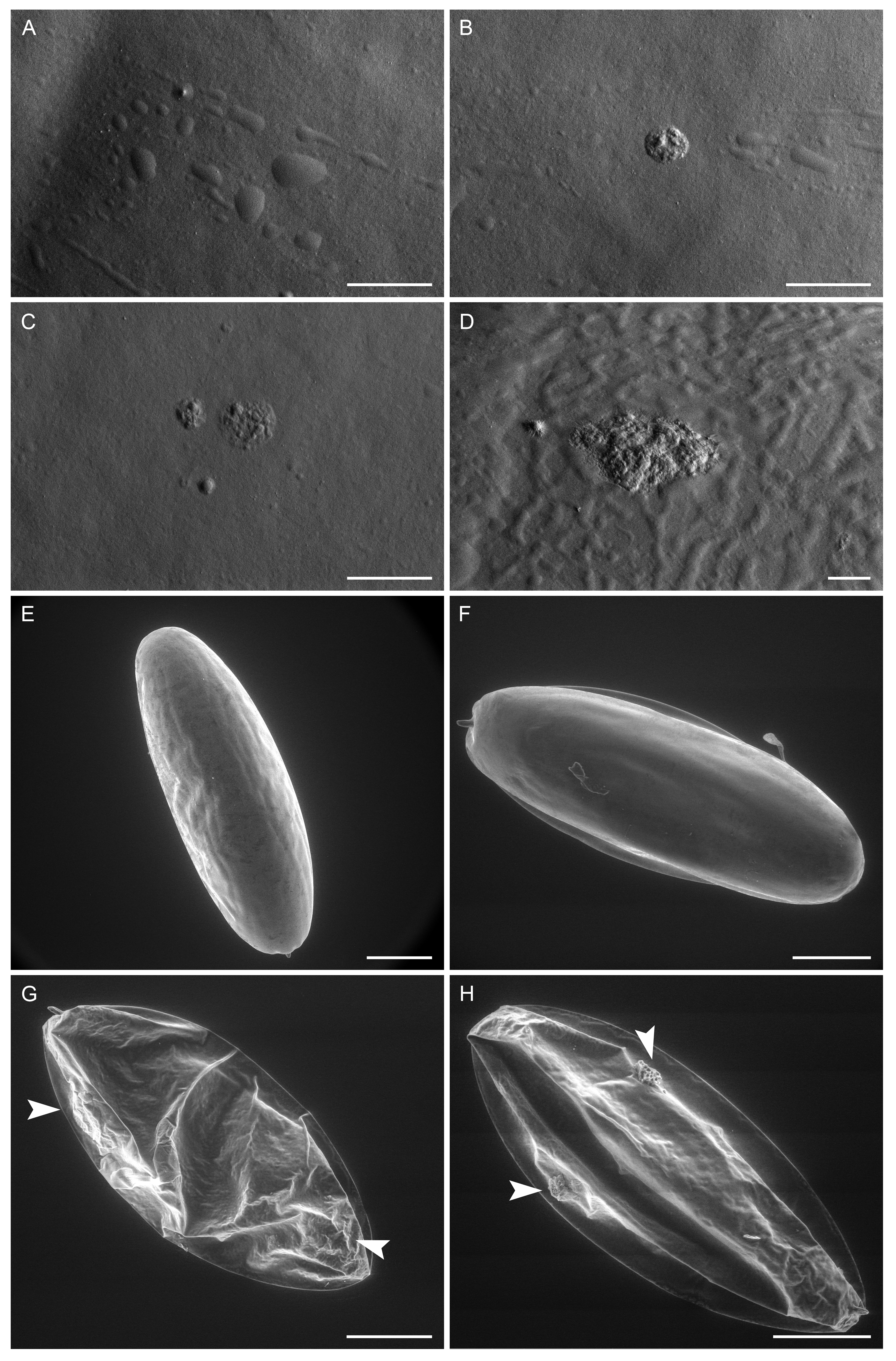

Supplement: S7 Fig — (A–D) Cryo-electron scanning images of hexane-washed egg surfaces sequentially depicting leakage of egg contents through the vitelline membrane taken on Helios. The vitelline membrane distends at weak spots (A), the egg contents then gradually permeate through the distended membrane forming small droplets (B, C), and eventually these droplets merge to form larger droplets (D). (E–H) Low-vacuum electron scanning images (taken on Quanta at low vacuum) of dechorinated eggs laid by transgenic mutant flies with ablated oes (oe−) that were either self-crossed or crossed with wild-type flies (oe+): ♂oe+ × ♀oe+ (E), ♂oe− × ♀oe+ (F), oe+ × ♀oe− (G), and ♂oe− × ♀oe− (H). Egg leakage was only observed in oe− mutant motherhood eggs (arrowheads in G and H); furthermore, these eggs collapsed under reduced pressure more rapidly than eggs with oe+ motherhood (E and F). Scale bar in panels (A–D) = 5 μm, and in panels (E–H) = 100 μm. oe, oenocyte (TIF) [file pbio.2006012.s007.tif]
