# Supplementary material for: Drosophila melanogaster cloak their eggs with pheromones, which prevents cannibalism
Source: PLoS Biol. 2019 Jan 10;17(1):e2006012. doi: 10.1371/journal.pbio.2006012 (PMC6328083; doi:10.1371/journal.pbio.2006012)
Supplement: S1 Table — The compounds (alkenes and alkadienes) were identified in mass spectrum of wax layer’s hexane extract of intact eggs of wild-type flies. Only the monoisotopic peak (12C) of the highest abundance ion of an identified compound is indicated in the table. APPI FT-ICR MS, atmospheric pressure photoionization Fourier transform ion cyclotron resonance mass spectrometry (DOC) [file pbio.2006012.s008.doc]

| **Peak** | **Ion mass**  **(measured), Da** | **Ion mass**  **(theor.), Da** | **Mass error, (ppb)** | **Ion type** | **Chemical name,**  **(molecular formula)** |
| --- | --- | --- | --- | --- | --- |
| **1** | **311.29449** | **311.29446** | **-96** | **[M+H]^+^** | **11Z,11-Octadecen-1-ol-acetate,**  **(cVA; C_20_H_38_O_2_)** |
| 2 | 320.34375 | 320.34377 | 62 | M^+^˙ | 7,11-Tricosadiene, (7Z,11Z)-,  (C_23_H_44_) |
| **3** | **322.35940** | **322.35942** | **62** | **M^+^**˙ | **7Z-Tricosene,**  **(C_23_H_46_)** |
| **4** | **348.37505** | **348.37509** | **115** | **M^+^**˙ | **7Z, 11Z-Pentacosadiene,**  **(C_25_H_48_)** |
| **5** | **376.40639** | **376.40635** | **-106** | **M^+^**˙ | **7,11Z-Heptacosadeine,**  **(C_27_H_52_)** |
| 6 | 378.32805 | 378.32810 | 132 | M^+^˙ | (22E)-3,5-Cycloergosta-6,8(14),22-triene, (C_28_H_42_) |
| 7 | 384.33872 | 384.33867 | -130 | M^+^˙ | Desmosterol,  (C_27_H_44_O) |
| 9 | 396.33873 | 396.33867 | -151 | M^+^˙ | Ergosterol,  (C_28_H_44_O) |
| **10** | **404.43770** | **404.43765** | **-124** | **M^+.^** | **7,11Z-Nonacosadeine,**  **(C_29_H_56_)** |
| 11 | 404.40131 | 404.40127 | -99 | M^+^˙ | Spiro[13,14]octacosane-15-one, (C_28_H_52_O) |
| 12 | 410.39078 | 410.39070 | -195 | M^+^˙ | Squalene,  (C_30_H_50_) |
| 12 | 426.38568 | 426.38562 | -141 | M^+^˙ | Lanosterol,  (C_30_H_50_O) |
| **13** | **464.42247** | **464.42240** | **-151** | **M^+.^** | **(11Z,19Z)- (3-acetoxy-11,19-octacosadien-1-ol),**  **(CH503; C_30_H_56_O_3_)** |
