# Supplementary material for: Drosophila melanogaster cloak their eggs with pheromones, which prevents cannibalism
Source: PLoS Biol. 2019 Jan 10;17(1):e2006012. doi: 10.1371/journal.pbio.2006012 (PMC6328083; doi:10.1371/journal.pbio.2006012)
Supplement: S2 Table — C1, ♂oe+ × ♀oe+; C2, ♂oe− × ♀oe−; C3, ♂oe− × ♀oe+; and C4, ♂oe+ × ♀oe−. The table shows the intensities (SNRs) of four major (the highest abundance) peaks (ion types) of the target compound identified in the corresponding mass spectra. Intensities were obtained from averaging of three mass spectra of each hexane extract acquired with separate runs. The sum of intensities of four major peaks of each compound were used to plot the histogram (Fig 2B). APPI FT-ICR MS, atmospheric pressure photoionization Fourier transform ion cyclotron resonance mass spectrometry; oe, oenocyte; SNR, signal-to-noise ratio (DOC) [file pbio.2006012.s009.doc]

| **Compound /**  **Ion type** | | **Ion**  **mass (theor.), Da** | **RMS mass error, ppm** | | | | **Intensity (signal-to-noise ratio)** | | | |
| --- | --- | --- | --- | --- | --- | --- | --- | --- | --- | --- |
|  |  |  | **(C1)** ♂oe^+^× ♀oe^+^ | **(C2)** ♂oe^-^× ♀oe^-^ | **(C3)** ♂oe^-^× ♀oe^+^ | **(C4)** ♂oe^+^× ♀oe^-^ | **(C1)** ♂oe^+^× ♀oe^+^ | **(C2)** ♂oe^-^× ♀oe^-^ | **(C3)** ♂oe^-^× ♀oe^+^ | **(C4)** ♂oe^+^× ♀oe^-^ |
| **CVA, (C_20_H_38_O_2_)** | [M+H-H_2_]^+^ | 309.27881 | 0.38 | 0.32 | 0.35 | 0.26 | 2.4±0.2 | − | 1.7±0.2 | 3.8±0.7 |
|  | [M+H]^+^ | 311.29446 |  |  |  |  | 20.8±2.6 | 6.1±0.2 | 5.1±0.6 | 40.9±9.5 |
|  | [M+H-2H_2_+H_2_O]^+^ | 325.27372 |  |  |  |  | 1.6±0.2 | − | 1.7±0.5 | 3.5±0.7 |
|  | [M+H-H_2_+H_2_O]^+^ | 327.28937 |  |  |  |  | − | − | − | 1.6±0.1 |
| **7Z-T, (C_23_H_46_)** | [M+H-H_2_]^+^ | 321.35158 | 0.32 | 0.66 | 0.52 | 0.35 | 2.4±0.1 | 1.2±0.1 | 1.7±0.1 | 4.0±0.7 |
|  | M^+^˙ | 322.35940 |  |  |  |  | 2.6±0.4 | 1.9±0.1 | 1.8±0.1 | 4.8±0.8 |
|  | [M+H-2H_2_+H_2_O]^+^ | 337.34649 |  |  |  |  | 1.6±0.2 | 1.3±0.2 | 2.2±0.5 | 3.8±0.4 |
|  | [M+H-H_2_+H_2_O]^+^ | 339.36214 |  |  |  |  | 1.9±0.2 | 1.7±0.3 | 2.4±0.2 | 3.8±0.4 |
| **7,11-HD, (C_27_H_52_)** | [M+H-H_2_]^+^ | 375.39853 | 0.91 | 0.50 | 0.87 | 0.91 | 3.8±0.1 | 1.3±0.2 | 6.3±1.0 | 2.8±0.1 |
|  | M^+^˙ | 376.40635 |  |  |  |  | 6.3±0.9 | 1.8±0.3 | 11.7±1.4 | 5.0±0.3 |
|  | [M+H-2H_2_+H_2_O]^+^ | 391.39344 |  |  |  |  | 2.6±0.2 | − | 5.0±0.4 | 2.6±0.1 |
|  | [M+H-H_2_+H_2_O]^+^ | 393.40909 |  |  |  |  | 1.2±0.1 | 1.2±0.1 | 2.2±0.1 | 1.6±0.2 |
| **7,11-ND, (C_29_H_56_)** | [M+H-H_2_]^+^ | 403.42983 | 0.32 | 0.80 | 0.71 | 0.80 | − | − | 2.6±0.1 | 1.5±0.1 |
|  | M^+^˙ | 404.43765 |  |  |  |  | 2.2±0.5 | 1.1±0.1 | 5.5±0.9 | 2.5±0.2 |
|  | [M+H-2H_2_+H_2_O]^+^ | 419.42474 |  |  |  |  | 1.2±0.6 | − | 1.9±0.6 | − |
|  | [M+H-H_2_+H_2_O]^+^ | 421.44039 |  |  |  |  | − | 1.7±0.2 | 1.9±0.4 | 1.5±0.2 |
